# Temperature-Dependent Intensity Modulated Two-Photon Excited Fluorescence Microscopy for High Resolution Mapping of Charge Carrier Dynamics

**DOI:** 10.1021/acsphyschemau.3c00013

**Published:** 2023-07-07

**Authors:** Qi Shi, Pushpendra Kumar, Tönu Pullerits

**Affiliations:** † The Division of Chemical Physics and NanoLund, Lund University, Box 124, 22100 Lund, Sweden; ‡ Department of Physics, Kirori Mal College, University of Delhi, Delhi 110007, India

**Keywords:** MAPbBr_3_ perovskite, intensity modulation technique, first-order recombination, second-order recombination, phase transition

## Abstract

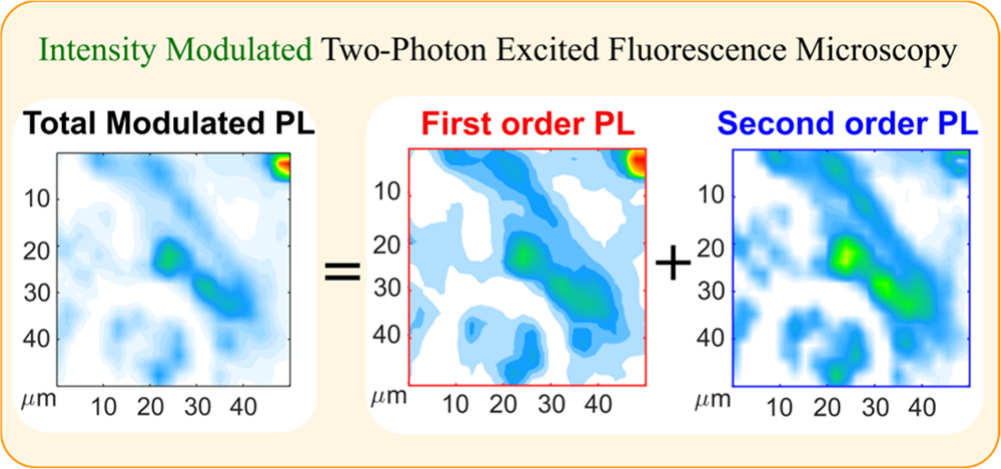

We present a temperature-dependent intensity modulated two-photon excited fluorescence microscopy technique that enables high-resolution quantitative mapping of charge carrier dynamics in perovskite microcrystal film. By disentangling the emission into harmonics of the excitation modulation frequency, we analyze the first and second order charge carrier recombination processes, including potential accumulation effects. Our approach allows for a quantitative comparison of different emission channels at a micrometer resolution. To demonstrate the effectiveness of the method, we applied it to a methylammonium lead bromide perovskite microcrystal film. We investigated the temperature-dependent modulated imaging, encompassing the exciton dissociation-association and charge carrier trapping-detrapping equilibrium. Additionally, we explored the potential freezing out of traps and the phase transition occurring at low temperatures.

## Introduction

For about a decade now, significant research activities have been focused on solution-processed hybrid lead halide perovskite semiconductors, for their promise in optoelectronics, including photovoltaics (PV),^1−9^ light-emitting diodes (LED),^10−15^ and lasers.^16,17^ All these applications are enabled by the beneficial properties of the material, such as strong optical absorption, high carrier mobility, long carrier lifetime, and high electron–hole diffusion length.^1,2^ The simple solution-based processing is prone to result in defects and structural inhomogeneities. Interestingly, the defects do not seriously deteriorate the critical characteristics of this material. These characteristics are all closely related to the carrier recombination processes. Clearly, understanding the impurity effects on the charge-carrier recombination dynamics with sufficient spatial resolution comparable to the structural inhomogeneities is the key for further improvements of such materials. The optoelectronic properties of MAPbBr_3_ perovskite exhibit a pronounced temperature dependence^18,19^ and undergo a phase transition at about 150 K shifting from the tetragonal to orthorhombic structure.^18^ Investigating the charge carrier recombination dynamics within this transition region provides valuable insights into impurities and their correlation with structural changes within the material.

Various spectroscopies have been utilized to follow the charge-carrier recombination dynamics, including photoluminescence (PL), transmittance, transient absorption, and optical pump-optical probe and terahertz (THz) probe spectroscopies.^3,20−22^ Because of morphological disorder, the importance of spatial resolution in the studies of charge carrier dynamics has been recognized^23−26^ and pronounced spatial segregation of the free charge and exciton populations in perovskite grains at submicrometer scale was demonstrated.^27^ Such effects would be averaged out if using traditional ensemble spectroscopy. Therefore, in studies of such materials, it is important to develop and apply techniques which can distinguish different elementary excitations and the related processes with sufficient spatial resolution.

Intensity modulation technique has been earlier used in two-photon excited fluorescence microscopy to spatially resolve the first-order and second-order processes in MAPbBr_3_ crystal at the micrometer scale.^5^ This technique utilizes two-photon excitation, which improves spatial resolution and enables deep material excitation.^28^ In addition, the utilization of intensity modulation techniques allows for the disentanglement of various emission processes, including both first and second-order recombination, in a manner that was previously unattainable with conventional methods. Despite these advancements, achieving a quantitative comparison between the excitations involved in first-order and second-order recombination has remained a challenging task until now. A comprehensive analysis of the fundamental charge carrier recombination processes in perovskite microcrystal film studies necessitates a quantitative assessment of the relaxation pathways of excitations. Furthermore, it is crucial to consider the potential accumulation of long-lived states induced by the high repetition rate laser pulses employed in these experiments, as they can significantly influence accurate quantitative analyses.^29^

In this work, we use spatially quantitatively resolved intensity modulated two-photon excited fluorescence microscopy to study temperature dependence of the charge carrier recombination dynamics. We apply a theoretical model which includes the charge accumulation effect. In this way we achieve quantitative comparison of different radiative channels (first-order, second-order, and accumulation-related PL) in MAPbBr_3_ perovskite microcrystal film with micrometer spatial resolution. With the help of a microscopic charge recombination dynamics model, various recombination processes are revealed. Via varying the temperature, the phase transition effect is investigated.

## Experimental Methods

### Instrumentation

The details of the experimental setup have been described elsewhere,^5,29−32^ and the schematic diagram is shown in Figure S1. Briefly, a Ti-sapphire oscillator (Synergy, Femtolasers) provides 20 fs excitation pulses at 70 MHz repetition rate centered at 790 nm. To compensate for the group velocity dispersion induced by the optical components, a chirped mirror pair was employed. Intensity modulation of the excitation was achieved by acousto-optic modulators (AOM) in the arms of a balanced Mach–Zehnder interferometer (MZI). The driving frequencies of the two AOMs were 55.00 and 54.95 MHz, respectively, resulting in a difference frequency of 50 kHz. One part of the output light from the interferometer was used as the reference to correct for the possible slow laser intensity fluctuations. The other part was sent to an inverted microscope and reflected by a dichroic mirror to the sample for two-photon excitation. A reflective objective with a numerical aperture of 0.65 was used to focus the laser beam onto the sample. Typically, the average power used for the two-photon excitation was about 10 mW. The laser focusing spot size was about 2 μm in diameter leading to two-photon excitation density of 7 × 10^13^ excitation/(cm^3^·pulse) (details provided in Supporting Information, section S2). The two-photon excited PL was collected by the same microscope objective. PL passed the dichroic mirror and was detected by an avalanche photodiode. A sample stage with a temperature-control unit (Linkam Scientific Instruments, LTS420E-P) was used to control the temperature between 113 and 298 K with a step of 20 K. The temperature values are rounded in this work.

### Sample Preparation

MAPbBr_3_ perovskite semiconductor was synthesized following the procedure described by Kojima et al.^33^ 21.12 g of MABr (Dyenamo, >98%, ∼0.01 mol) and 3.67 g of PbBr_2_ (Sigma-Aldrich, ≥98%, ∼0.01 mol) were dissolved in 10 mL of *N*,*N*-dimethylformamide (Sigma-Aldrich, ≥99%) to obtain a precursor. The glass substrates were washed by acetone in an ultrasound machine. 100 μL of precursor was pipetted on the glass substrate and annealed at 80 °C until the complete evaporation of solvent, resulting in the formation of bulk crystals. To ensure the sample conductivity during the SEM measurements, a 5 nm Pd/Pt layer was coated on the perovskite crystals using a Q150t ES metal sputter coater. The SEM image (Figure S2) is present in the SI.

### Charge Carrier Recombination and Fluorescence

Fluorescence intensity is directly related to the charge carrier recombination processes which are commonly described by a generic model in terms of the orders of carrier concentration or population *n*:^3,4,34^
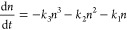
1Here, *k*_1_ is the rate constant associated with the first-order process like Shockley-Read-Hall (SRH) trap mediated recombination and geminate recombination of the electron–hole pairs. *k*_2_ is the second-order charge-carrier recombination rate constant, which depends on the square of the population—for example multiplication of the population of electrons and holes in nongeminate recombination. The process is often described by a Langevin model where Coulomb-coupled electron and hole recombine if they come close enough. It is generally accepted that in hybrid perovskites the second order recombination rate *k*_*2*_ is far slower than what is predicted by the Langevin theory.^34^ Most likely the electron and hole do not recombine by their first encounter and the recombination can be described via temperature-dependent Saha–Langmuir (S-L) equilibrium between the free charges and excitons where the latter can efficiently emit light and thereby recombine. Such process depends quadratically on the carrier concentration^18,19,35^ and the resulting exciton emission follows the second-order dependence. The third order rate *k*_3_ represents the Auger recombination in which the energy of the electron–hole pair is transferred to a third particle, either an electron, a hole or an exciton. The resulting highly excited hot charge carriers rapidly cool down by relaxing to the band edge.^36,37^ The possible higher order terms (>3) provide negligible contribution, and recombination in semiconductors is usually described by the above three components.^34,38^ Since the Auger recombination takes place at high charge carrier densities (≈10^16^ to 10^17^ cm^–3^), this component too is insignificant for our two-photon excitation conditions (10^13^ cm^–3^) and can be left out.

In experiments where the excitation pulse repetition rate is high, the long-lived states (trap states, e.g.) can be accumulated.^29^ Such accumulation effects can lead to additional nonlinearities even if otherwise the recombination is of the first order. In the following article we analyze the details of the intensity modulated two-photon excited fluorescence for the first and second order processes taking into account also the possible accumulation effects.

### Intensity Modulated Two-Photon Excited Emission for the First and Second Order Processes

In the MZI unit, each AOM acts as a Bragg cell with slowly propagating longitudinal acoustic wave oscillating at a frequency Ω_*i*_ in the medium. The acoustic wave influences the optical field via the grating it generates in the refractive index of the medium. The light (laser pulse) speed is much higher than the velocity of the acoustic wave. Therefore, for a given laser pulse at time *t*, the acoustic wave is standing with the phase Ω_*i*_*t* which is imparted to the laser pulse passing the Bragg cell. The pulse will obtain a phase shift Ω_*i*_*t* which is varying with time.

The electric field *E* of the optical waves of the two laser pulses after passing the AOMs can be expressed as
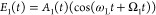
2
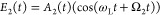
3where *A*_1_ and *A*_2_ are the pulse envelopes and *ω*_L_ is the optical frequency of the pulses (379 THz @ 790 nm). Ω_1_ and Ω_2_ are the driving frequencies of AOMs (55 and 54.95 MHz, respectively) which are much lower than the optical frequency. The total electric field of the phase-modulated pulses can be written as
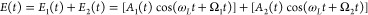
4In the experiment the two pulses are made coincident in time while the phase difference of the pulses changes with time. The laser beam intensity is proportional to the square of the field. Consequently, the intensity of the pulse train is modulated at the difference of the phase modulation frequencies Ω = Ω_1_ – Ω_2_ = 50 kHz:
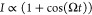
5We call this the basic harmonic or the first harmonic. The probability of two-photon absorption is proportional to the square of the light intensity.^28^ Therefore, the excitation concentration or, in other words, population, has, besides the first harmonic, even the second harmonic frequency present.

For the first-order recombination process, the PL emission is proportional to the population of the excitations. By taking eq 5 to the second power we find that the total signal consists of a constant component, the first harmonic, and the second harmonic with the ratio between them 3:4:1, respectively.

The second-order recombination PL signal is proportional to the square of the population. This signal has more components than the first order PL—besides the constant part, four harmonics appear. These five signal components relate to each other as 35:56:28:8:1.

Here a comment about the constant component of the signal. The constant signal can arise from all three mechanisms: first order and second order recombination, as well as accumulation-related nonlinearities. In the above analyses of the first and second order signals, we were able to quantify the constant component (as shown in Table 1). However, for the analyses of the accumulation-related nonlinearities (as described in the next section), separating the constant part is not straightforward. Besides, there always is experimental background. Therefore, in the following analyses, we analyze only the modulated part of the signal.

**Table 1:** Decomposition of the Total Modulated PL Emission Amplitudes of Four Harmonics (A1H, A2H, A3H, and A4H) in Terms of the First-Order PL ***r***_**1**_, Second-Order PL ***r***_**2**_, and AE (Accumulation Effect) PL ***r***_**1***A*_

total modulated PL	A1H	A2H	A3H	A4H
First-order PL	*r*_1_	*r*_1_		
Second-order PL	*r*_2_	*r*_2_	*r*_2_	*r*_2_
AE PL	*r*_1*A*_	*r*_1*A*_*R*_21_	*r*_1*A*_*R*_31_	*r*_1*A*_*R*_41_

We concentrate on four harmonic frequencies with the aim to disentangle the various origins of the modulated emission like the first order and second order recombination processes. For one unit of the first-order PL, the amplitude of the modulated signal is . Analogously, for one unit of the second order PL, the modulated signal is . The analyses develop further the previous work^5^ providing a detailed quantitative comparison of the different PL contributions.

### Nonlinearities Due to the Accumulation Effect (AE)

In the case of a high laser repetition rate, the ground state recovery time due to the trap-mediate recombination processes can be longer than the interval between the laser pulses (in our case *t*_0_ ≃ 14.25 ns) and the accumulation-related nonlinear effects can take place. Such nonlinearities can originate from the ground state depletion and can lead to additional contributions to the harmonics.^29,39^ In the following we present analyses of such accumulation effect in the case of first-order recombination process after two-photon excitation.

The time evolution of the excited state population *P*(σ) can be described by the kinetic equation
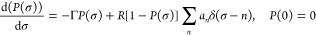
6where  is the time in the units of pulse-to-pulse time interval *t*_0_.  is the amount of decay during the pulse interval, and τ is the ground state recovery time. *R* is the amount of excitation by one pulse in case of fully recovered ground state and [1 – *P*(σ)] accounts for the depletion of the ground state due to the accumulation. The first term at the right-hand side of eq 6 describes the population decay bringing the system back to the ground state. The second term describes the probability of two-photon excitation by the intensity modulated pulses with *a*_*n*_ = (1 + cos (Ω*t*))^2^ where *n* refers to the *n*^th^ pulse in the laser pulse train, and the delta function warrants timing of the pulses so that *t* = *nt*_0_.

**Figure 1:**
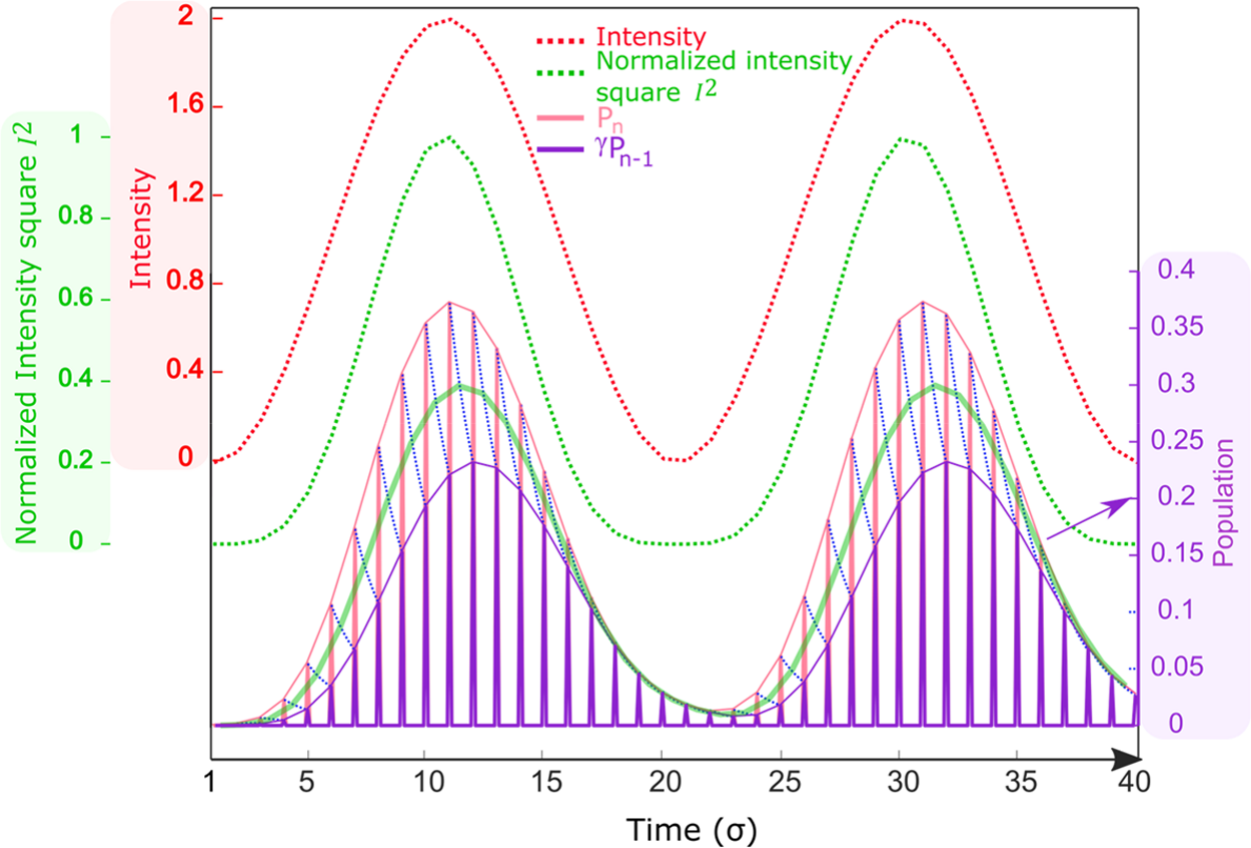
Illustration of the recurrence equation of accumulation (eq 7). The time on *X*-axis is counted in the pulse intervals—the whole scale of two modulation periods corresponds to 40 pulses. For the clarity of the simulations, this number has been chosen much smaller than in real experiment where ∼3000 pulses excite the sample during the two intensity modulation periods. The red dotted line provides the envelope of the modulated laser radiation intensity (1 + cos(Ω*t*)), and Ω = (Ω_1_ – Ω_2_) is the intensity modulation frequency. The green dotted line is the normalized intensity square *I*^2^ (two-photon excited population) without the accumulation-related depletion effect. The other curves illustrate the accumulation effect. The population *P*_*n*_ directly after the *n*^th^ pulse is represented by the pink line. *P*_*n*_ is equal to the sum of the new excitation generated by the *n*^th^ pulse (pink) and the remaining excitation from the previous pulses, violet. The thin pink line joins the tops to illustrate the trend. The violet line is the remaining excitation from the previous pulses *γP*_*n*__–1_, and the thin violet line joins the corresponding tops. The thin blue dotted line represents the exponential decay of the population between two excitation pulses. The green line represents the averaged first-order recombination PL given by the integral over the time between two pulses. Clearly the green line has different shape compared to the population without the accumulation effect, the green dotted line, meaning that it has additional frequency components. In the simulations, γ is set to 0.62 (corresponds to the ground state recovery time 30 ns), *P*_0_ = 0, and *R*_0_ = 0.2.

From eq 6, the following recurrence relation can be obtained:
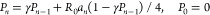
7γ = *e*^–Γ^ describes the exponential decay of the excitation, and *R*_0_ is the two-photon excitation probability by maximum intensity of a pulse from fully recovered ground state. 1/4 accounts for the maximum value of *a*_*n*_ = 4. The first term of the right-hand side of eq 7 represents part of the previous pulse excitation which has still not decayed back to the ground state. The second term represents the new excitation by the n^th^ pulse. Equation 7 is illustrated in Figure 1. A more detailed description of the accumulation effect is provided in section S3.

### Total Modulated Signal

Summary of the all above analyzed contributions to the modulated PL emission is presented in Table 1.

In the frame of the model which consists of the first-order PL (*r*_1_), accumulation effect PL (*r*_1*A*_), and the second order PL (*r*_2_), we express the modulated part of the experimental data as four harmonic components:
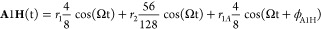
8
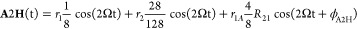
9
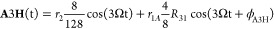
10
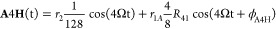
11

We can see that the first and second harmonic (A1H, A2H) obtain contributions from all three mechanisms. The third and fourth harmonic (A3H, A4H) originate from the second order PL and from the accumulation effects, while there is no contribution from the first order PL at these frequencies. The *r*_1_, *r*_2_, and *r*_1*A*_ correspond the total PL (the sum of the modulated and constant signal) from the three mechanisms. *R*_21_, *R*_31_, and *R*_41_ are the PL amplitude ratios of the harmonic components due to the accumulation-related nonlinear effects. While the first and the second order emission does not lead to any phase shift, the accumulation-related nonlinearity can lead to a such shift denoted ϕ_A1H_, ϕ_A2H_, ϕ_A3H_, and ϕ_A4H_, for the four harmonics. For the detailed expressions, see section S3.

**Figure 2:**
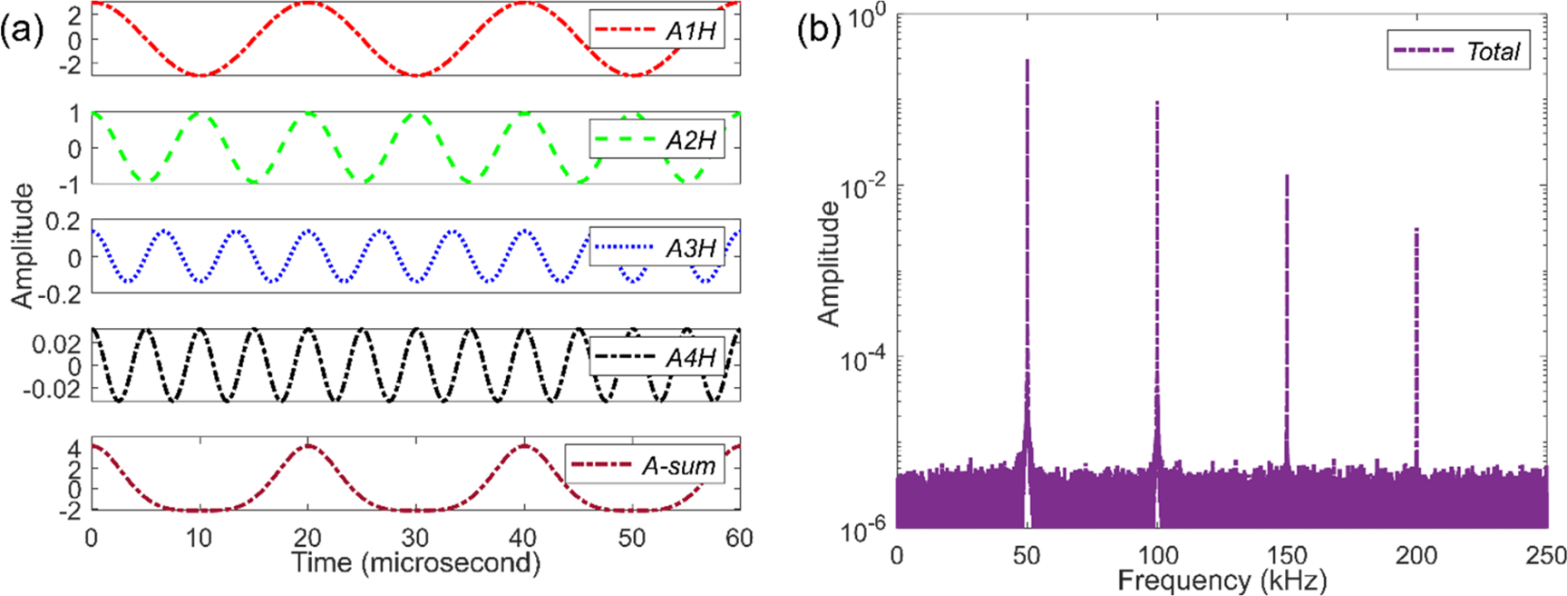
(a) Simulated time-domain signals of the four harmonics (A1H, A2H, A3H, and A4H) and the total signal (A-sum), consisting of the first-order recombination, second-order recombination, and first-order recombination with accumulation effect processes. (b) The frequency-domain signal obtained via the Fast Fourier transform (FFT) of the time-domain signal (0.1 s for 2 MSa/s sampling rate). A small noise was added in time domain before FFT to realistically mimic the experimental data.

As an example, simulated time and frequency domain signals are shown in Figure 2. The *r*_1_, *r*_2_, and *r*_1A_ are set to 1, and the corresponding harmonics ratios follow Table 1. The excitation probability *R*_0_ is set to 0.01, and the ground state recovery time is 200 ns. The details are provided in section S4.

### Experimental Results, Analyses, and Discussion

**Figure 3:**
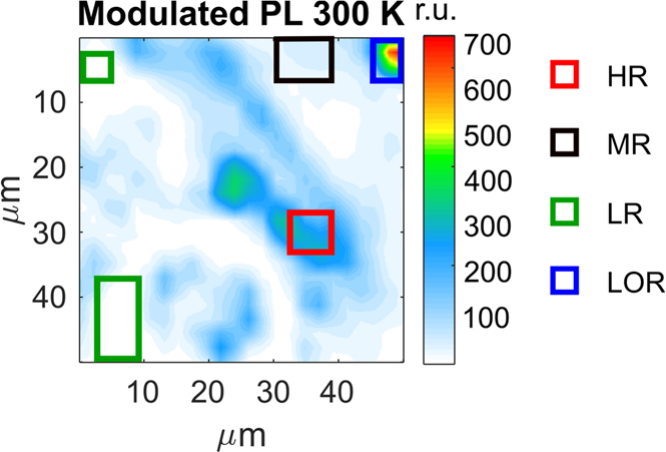
Assignment of the regions based on the modulated PL emission intensity at room temperature. HR: red, high PL region. MR: black, medium PL region. LR: two green rectangles, low PL region. LOR: blue, high emission localized spot region.

As a demonstration, we analyze MAPbBr_3_ perovskite microcrystal by the spatially resolved intensity modulated two-photon excited fluorescence microscopy for quantitative analysis of excitation dynamics and the related recombination processes. The modulated PL intensity varies significantly over the investigated 48 × 48 μm^2^ region of the sample, see Figure 3. We define three distinct emission intensity levels at room temperature, and the corresponding regions are denoted as high (HR), medium (MR), and low PL region (LR) marked with red, black, and green rectangles, respectively. Besides, there is a small region with very high PL intensity which we call the localized spot region (LOR). In the following, we perform detailed comparative analyses of these regions.

The frequency components of the time-domain experimental data are obtained by using the FFT with MATLAB software. We point out that the data used here are analogous to that in our previous work.^5^ Here the temperature range is broader and the analyses is more detailed allowing quantitative comparison of the different PL contributions.

The amplitudes and phases of the four harmonics were fitted using the relations between the components in Table 1 and the eqs 8–11. Nonlinear least-squares analysis (Trust-Region algorithm) as implemented in MATLAB software, was applied. The fitting has four independent parameters—PL amplitudes originating from the three mechanisms *r*_1_, *r*_2_, *r*_1*A*_, and the ground state recovery time τ. The error analyses of the fit are provided in section S5.

**Figure 4:**
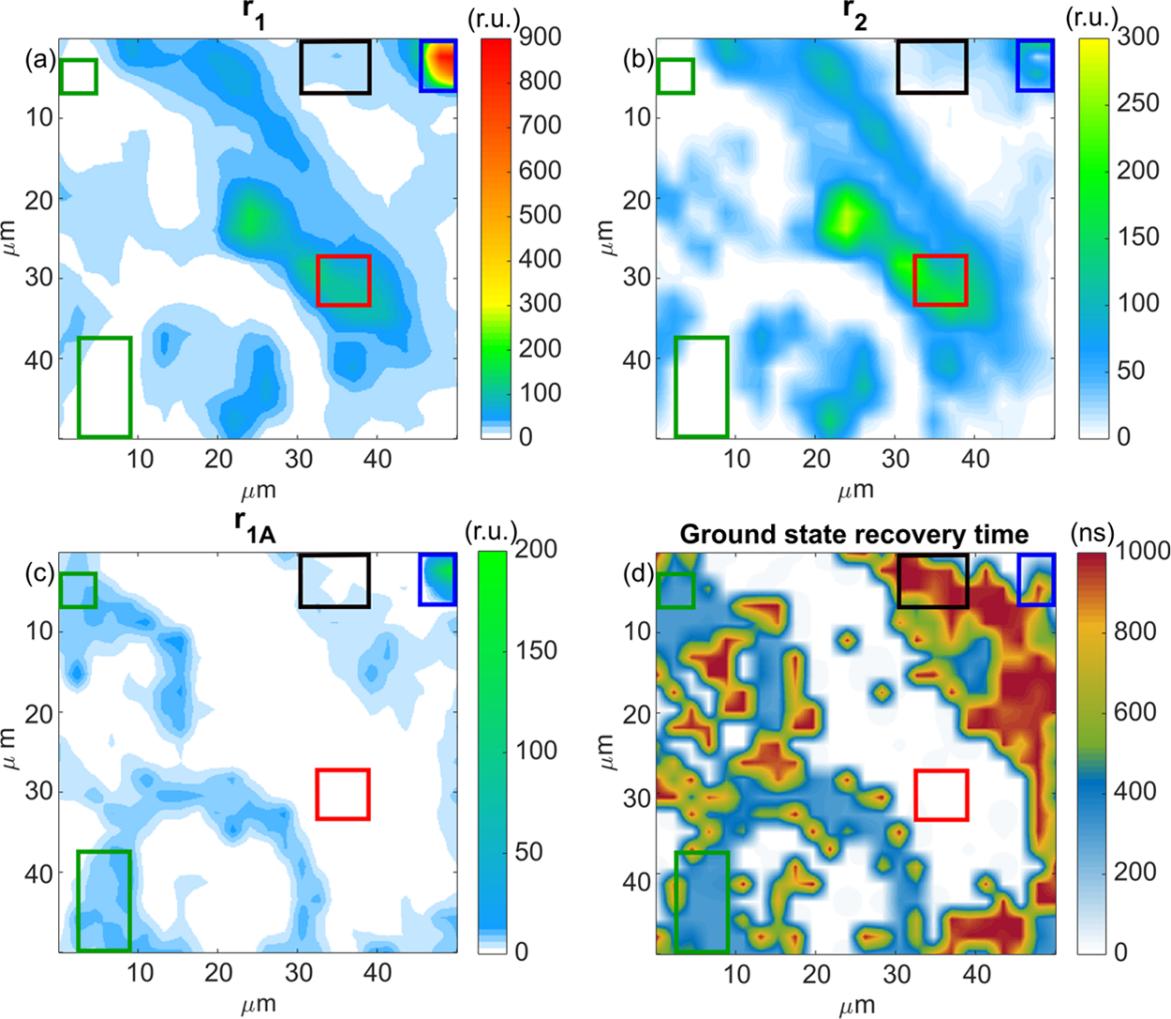
Micrometer scale imaging of the three components of the PL in the MAPbBr_3_ perovskite crystal at 300 K. (a) First-order PL (*r*_1_), (b) second-order PL (*r*_2_), (c) accumulation-related PL (*r*_1*A*_), and (d) the ground state recovery time τ of the accumulation effect. Four regions (high PL region, medium PL region, low PL region, and localized spot region) are marked with red, black, green, and blue rectangles, respectively.

The maps of the PL amplitudes from different charge carrier recombination mechanisms (first-order *r*_1_, second-order *r*_2_, accumulation effect *r*_1A_) and the accumulation related ground state recovery time at room temperature are shown in Figure 4.

It is obvious from the maps that the first-order PL and the second-order PL are quite well correlated. Another strong correlation is between the accumulation-related PL (*r*_1A_) and the ground state recovery time. At the same time, there is a clear anticorrelation between the first correlated pair and the accumulation related PL, which shows that the excited state population (electron–hole pairs or excitons) recombines either through the first-order/second-order PL emission or through the accumulation effect PL with longer ground state recovery time.

The total modulated PL emission intensity due to the three mechanisms (*r*_1_ + *r*_2_ + *r*_1A_) for high, medium, and low PL region, and the localized spot relate to each other as 1:0.2:0.05:3.2 (see section S8). Clearly, the emission intensities cover a very broad range. The significantly brighter localized PL emission spot may originate from morphology-related large two-photon absorption coefficient or significantly reduced deep trap concentration.^40−42^

**Figure 5:**
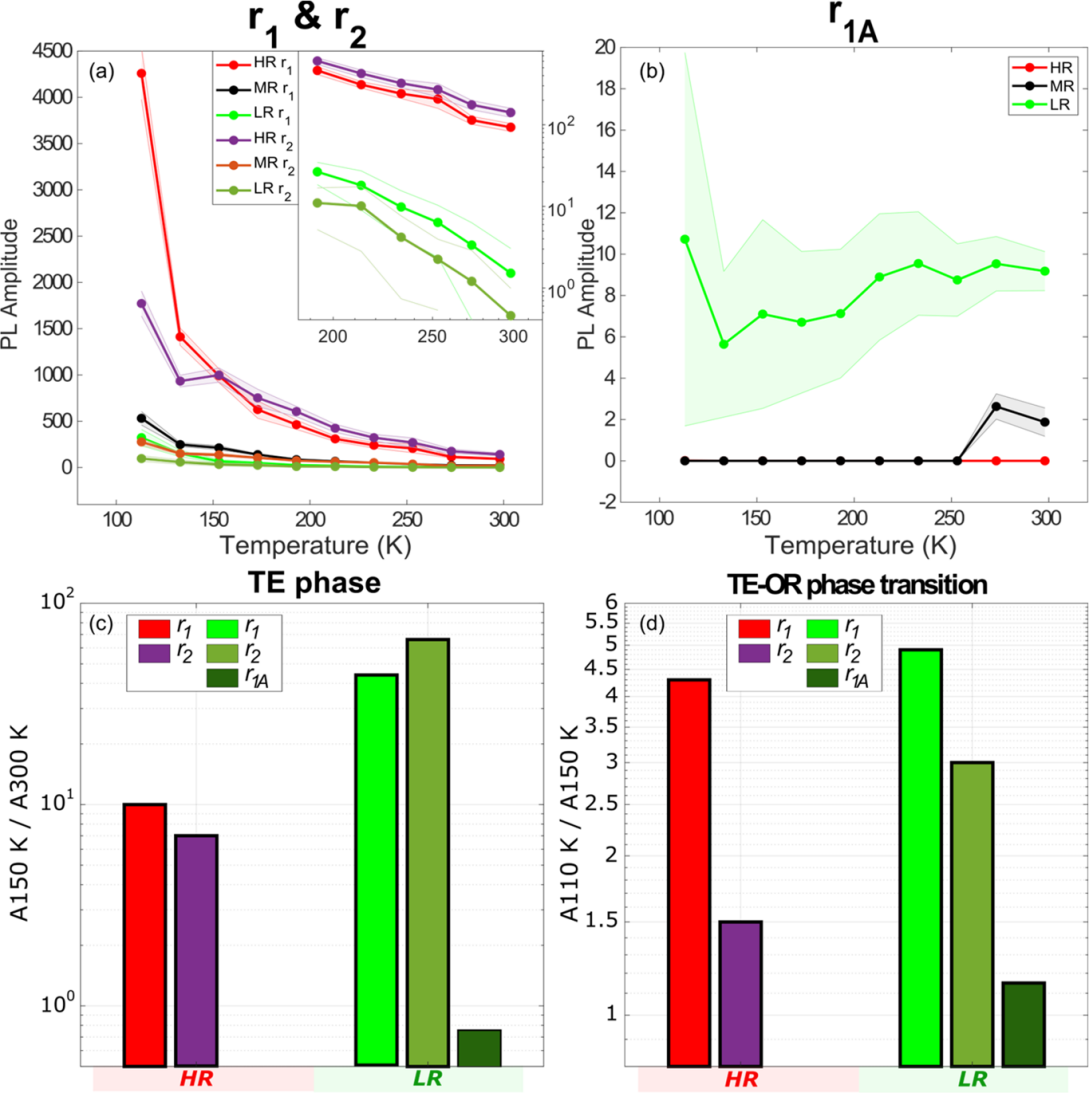
Temperature dependence of the different emission contributions in the three characteristic regions of the PL intensity. (a) First-order PL (*r*_1_) and second-order PL (*r*_2_); (b) accumulation effect PL (*r*_1__A_). The inset in figure a shows the *r*_1_ and *r*_2_ PL in HR and LR increase with temperature decreasing from 300 to 200 K. The shaded area represents half of the standard deviation (SD). The SDs are calculated based on the 9, 16, and 22 pixels in the HR, MR, and LR, respectively. (c) Ratios of PL component amplitudes at 150 K over the PL emission at 300 K from HR and LR (TE phase). (d) Same PL component amplitude ratios as (c) for the emission at 110 K over the emission at 150 K from HR and LR. Between 110 and 150 K phase transition takes place.

The same analysis of the modulated emission components as above was carried out for the temperature range from room temperature down to 110 K. The three regions of different PL intensities (HR, MR, and LR Figure 5) behave qualitatively differently from the LOR region (Figure S8). For the HR, MR, and LR regions, the first-order and second-order PL emission increase with temperature decreasing (Figure 5a). A significant charge accumulation effect is present in the LR, see Figure 5b. The corresponding ground state recovery times for the different regions are shown in Figure S9. Interestingly, the recovery time in MR jumps from very low values to about microseconds when approaching room temperature. In HR the accumulation-related emission is negligible. In MR it is near zero at low temperature while at close to room temperature it slightly rises.

We calculate the ratios of PL emission components which are related to the different mechanisms *r*_1_, *r*_2_, and *r*_1A_ at different temperatures. In one case we use 300 and 150 K, both corresponding to the TE phase, but the temperature difference is two times. The other pair of temperatures is 110 and 150 K; between these temperatures the phase transition from TE to OR occurs, while the temperature difference is relatively small. In Figure 5c we show the ratios of the PL component amplitudes  for HR and LR.  for the same regions are shown in Figure 5d. We can see that while the purely temperature related ratio, , for the first (*r*_1_) and second order (*r*_2_) PL components is quite high in both analyzed regions, the difference between the regions is large. In the LR region the ratio is about 50 for the two components. While in the high PL region HR, the corresponding ratio is about 10. The same ratios for the temperature change involving the phase transition, , are significantly smaller. Remarkably, the two regions in this temperature range behave very similarly—in both cases the second order emission ratio is about a factor 2 lower than the first-order ratio. The accumulation related emission does not depend strongly on temperature.

## Discussion

**Figure 6:**
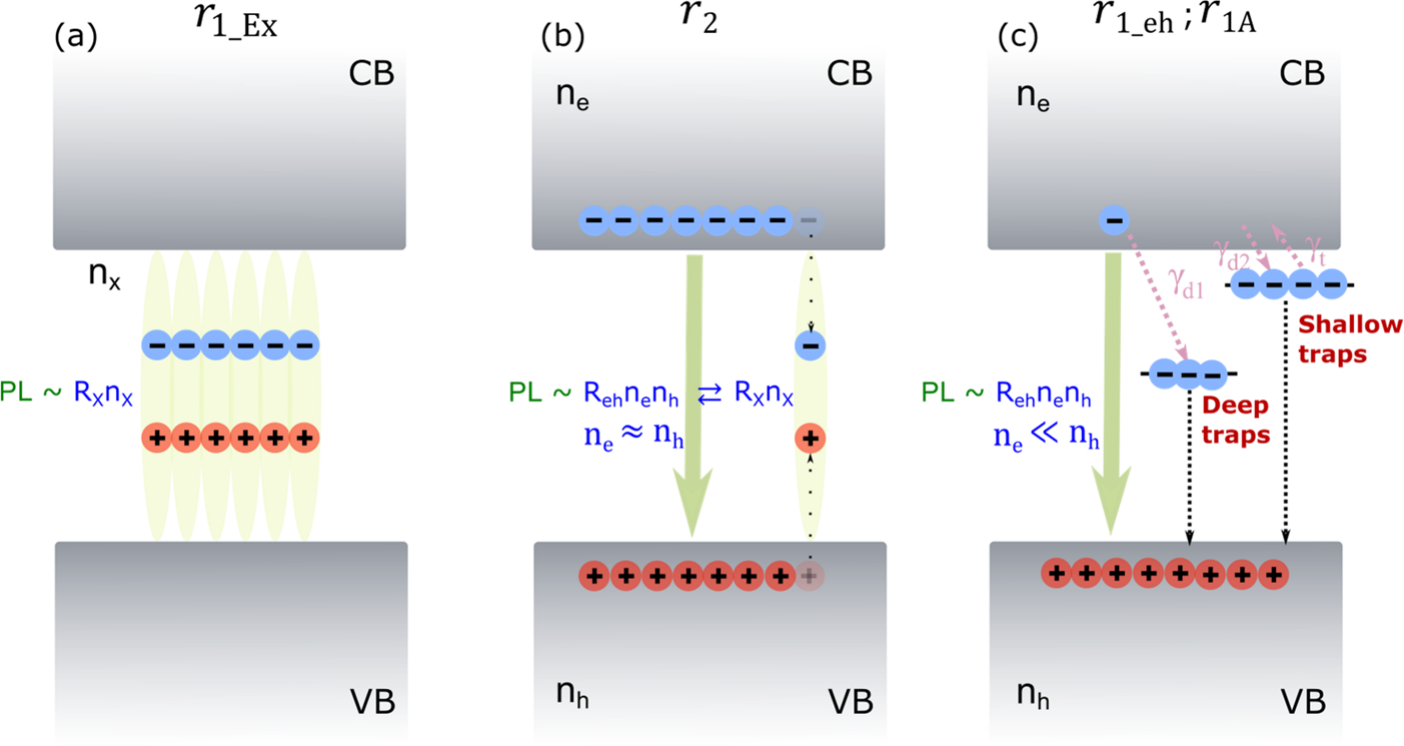
Schematics illustrating recombination mechanisms of perovskite microcrystal film. The conduction band and valence bands are denoted as CB and VB, respectively. The electrons in the CB and holes in the VB are denoted as *n*_e_, marked with light blue color, and as *n*_h_, marked with light red color, respectively. (a) The PL emission from the geminate e-h pair excitons *n*_*x*_ is of the first order and labeled as *r*_1_Ex_. (b) The PL emission from the electron–hole (e-h) pairs or excitons which are formed via the electron–hole nongeminate association to excitons, are second order and expressed as *r*_2_. Here the electron and hole populations are well balanced (nearly equal). (c) The PL emission from the unbalanced population (because of the electron traps, the electron population in the conduction band is much lower than the hole population in the valence band) of electron–hole (e-h) pairs is first order and is expressed as *r*_1_eh_ or *r*_1A_ in the case of charge accumulation effect. From the deep traps no detrapping of electrons back to the conduction band occurs. Such traps act as centers of nonradiative decay. From shallow traps at higher temperatures, electrons can be detrapped.

The above results can be explained with a general model described in Figure 6, where we relate the charge carrier dynamics and recombination to the first-order PL (*r*_1_), second-order PL (*r*_2_), and accumulation-related PL (*r*_1A_).

Right after the two-photon excitation by an intense laser pulse, high energy electron–hole (e-h) pairs are generated. Such initially hot carriers will relax (cool) to the band edge due to the carrier-phonon scattering.^18,19,37^ Since the two-photon excited charge carrier concentration is relatively low, excitons are likely formed from the geminate electron–hole pairs. These excitons can dissociate forming free electrons and holes. The widely used S-L description of PL usually assumes that only excitons emit.^43^ However, comprehensive modeling of bimolecular radiative recombination based on Elliott’s theory^44,45^ has shown that PL is a true reverse process of absorption and that even free charge carriers can emit. Importantly, Coulomb coupling can significantly enhance radiative recombination of the free electron–hole pairs.^45^ The quasi-balance between excitons *α*_*X*_ and the free electrons and holes *α*_free_ can be described by an S-L equilibrium model, which depends on the excitation density, exciton binding energy, and temperature.

Figure 6a depicts the first-order PL emission from the initial geminate e-h pairs and the corresponding excitons, denoted as *r*_1_Ex_. The excitons dissociate to electrons and holes. At longer time scale electrons and holes get mixed and if the electron population is comparable to the hole population, as illustrated in Figure 6b, the second-order PL emission from nongeminate electron–hole pairs or the corresponding excitons takes place and is denoted as *r*_2_. At longer times the first-order PL emission can emerge if the carrier population becomes heavily unbalanced, since the electron population becomes significantly lower than the hole population, as illustrated in Figure 6c. The first-order PL emission in this case is denoted *r*_1_eh_.

In the high emission region HR at room temperature the second-order emission *r*_2_ dominates over the first order emission. Even though the exciton binding energy is comparable to the room temperature *kT*, the corresponding equilibrium exciton population relative to the free charges is quite low because the low concentration of the charge carriers slows down the carrier association to the excitons (see Figure S10). Still the second order PL emission *r*_2_ mainly originates from the nongeminate electron–hole pairs or the corresponding excitons. Emission from the initial geminate e-h pairs and the corresponding excitons *r*_1_Ex_ exists but is short-lived and can only give a weak contribution. The first order emission at room temperature mainly originates from the disbalanced electron hole pair population emission *r*_1_eh_, see Figure 6. There are two main reasons. First, when the thermal energy is higher than the impurities’ ionization energy, such impurity related traps are activated and the electrons can be trapped by them.^21,46^ Such impurities are mainly due to the Frekel-type point defects, including vacancies, interstitials, and substitutions,^47,48^ denoted as shallow traps in Figure 6. The population of the electrons which are detrapped from the shallow trap states is much smaller than the population of the surrounding holes, therefore the radiative recombination of the detrapped electron and the surrounding holes is of the first order. Besides, with trapping, the disbalance of the electron and hole population means that the related PL becomes mostly of the first order as well.

As the temperature drops, both the first-order and second-order PL emission rise. The rise of the emission is readily explained by the stabilization of the excitons when the thermal energy *kT* becomes significantly smaller than the exciton binding energy. In this way not only the first-order exciton emission *r*_1_Ex_ from the initial geminate e-h pairs is increased, we also get more of the second order emission originating from the Saha–Langmuir balance of the nongeminate electron hole association and dissociation. Besides, when the thermal energy *kT* is lower than the ionization energy of the shallow traps, the population of the electron in the shallow trap states *n*__Tr__shallow_ and the holes *n*_h_ are decreasing due to the absence of the ionization process. As a result, the nonradiative recombination loss (γ_nonrad_*n*_Tr___shallow_*n*_h_ + γ_nonrad_*n*_Tr___deep_*n*_h_) decreases and the radiative first-order and second-order PL emission increases. Another obvious effect is the less efficient detrapping^49−52^ at lower temperatures which increases the trapped carrier population and the related unbalance of the carriers. In such unbalanced carrier conditions, the electron–hole pair association to excitons and the related PL becomes the first order process.

In Figure 5c, as the temperature decreases from RT to 150 K, the relative PL increase expressed by the ratio , is lower in the HR region than in the LR region. This suggests that the trap concentration is larger and/or the ionization energy of trap states^21^ is higher in the LR region.

When the temperature drops from 150 to 110 K, the phase transition occurs from the TE (high temperature) to OR (low temperature) structures.^19,53−56^ The ratio  for the *r*_1_ is 2–3 times larger than for the *r*_1_ in both HR and LR, see Figure 5d. This suggests that the intrinsic optical properties of the material changes which improves the first order emission more than the second order emission. The similar behavior in both HR and LR may show the fact that the phase transition induced photoluminescence behavior may not be strongly dependent on the morphology heterogeneity.

**Figure 7:**
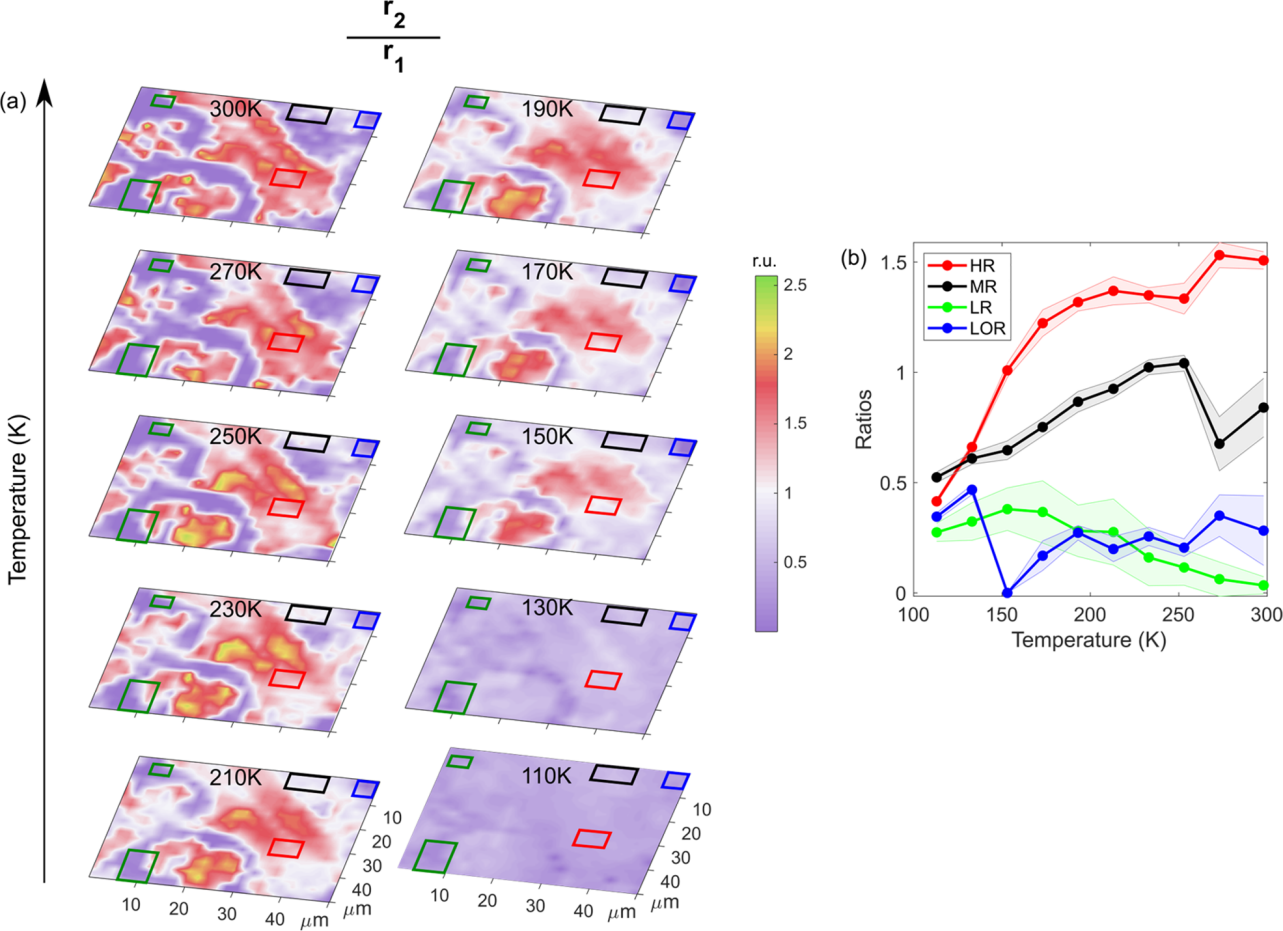
(a) Temperature-dependent spatially resolved SFR maps. (b) Temperature dependence of the SFR from the four characteristic regions.

From the discussion above we can see that *r*_1_ and *r*_2_ are correlated but are also clearly complementary to each other. To further analyze the mutual behavior of the second and first order PL, we combine them into a single parameter that we call second-to-first order ratio (SFR):
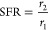
12Figure 7 shows SFR maps at different temperatures and the temperature dependence of the average SFR of the four distinct PL regions.

The most pronounced feature in the SFR representation is a clear change of the maps with the phase transition. While the maps above the phase transition temperature have high contrast that partially follows the earlier maps in Figure 4, the two maps below the phase transition temperature have almost no features. At room temperature, the SFR in the high PL region is significantly larger than the ratio in the low PL region. Clearly, efficient PL can be related to the dominating second order emission *r*_2_ compared to the first order emission. The latter is typically related to the traps either via direct unintentional doping related trap-mediated recombination of the electron–hole parts or as an origin to strongly unbalanced charge carrier population.

With temperature decreasing from room temperature to 150 K (temperature effect), the SFR ratio in the high PL region is decreasing while the SFR ratio in the low PL region is increasing. The former can be related to the increase of the first-order PL emission *r*_1_eh_ from the disbalanced electron hole pairs at low temperatures. The latter can be mainly related to the significant increase of the *r*_2_ at low temperature.

As temperature drops further from 150 to 110 K (phase transition effect), the SFR ratio in different regions behaves quite similarly. This suggests that the phase transition from TE to OR structure mostly changes the inherent optical properties of perovskite microcrystals similarly in all PL regions.

The PL maps of Figures 3, 4, and 7 have features which well match the usual grain size of the perovskite microcrystal films. Our interpretation is that the trap-rich regions with low PL intensity and dominating first order PL correspond to the interface or the region of the small grains, while the highly emitting regions with mainly second order PL come from the bulk of the perovskite grain. To verify this interpretation, such studies need to be combined with scanning electron microscopy. This would go beyond the scope of the current article and will be addressed in our upcoming work.

## Conclusions

We have presented a novel temperature-dependent intensity modulated two-photon excited fluorescence microscopy technique that allows for quantitative mapping of charge carrier recombination processes. By disentangling the emission into harmonics of the excitation modulation frequency, we have successfully achieved micrometer-resolution quantitative comparisons of various emission channels, including first-order recombination, second-order recombination, and recombination influenced by accumulation effects, in methylammonium lead bromide perovskite microcrystal films.

The new method offers several advantages for investigating the optoelectronic properties of materials. Our study has provided valuable insights into exciton dissociation–association, charge carrier trapping-detrapping equilibrium, and the potential freezing out of traps, as well as phase transitions at low temperatures in perovskite microcrystal films. These findings demonstrate the effectiveness of our approach in studying and understanding the intricate dynamics of charge carriers in semiconductor materials.

## Supporting Information Available

S1. Optical setup. S2. Excitation fluence calculation. S3. Accumulation effect in the first-order recombination process. S4. Intensity modulation spectroscopy model simulation. S5. The relative errors of A1H, A2H, A3H, and A4H for four-character regions performance at different initial given time scales and temperatures. S6. Temperature dependence of *r*_1_, *r*_2_, *r*_1A_, and τ in the localized spot region. S7. Temperature dependence of τ in the HR, MR, and LR. S8. Summarized results of the *r*_1_, *r*_2_, *r*_1A_, and τ for four-character regions at different temperatures. S9. Modeling of the fraction of free charges over the total excited state population. S10. Abbreviations in this paper (PDF)
