# Supplementary material for: Temperature-Dependent Intensity Modulated Two-Photon Excited Fluorescence Microscopy for High Resolution Mapping of Charge Carrier Dynamics
Source: ACS Phys Chem Au. 2023 Jul 7;3(5):467–76. doi: 10.1021/acsphyschemau.3c00013 (PMC10540292; doi:10.1021/acsphyschemau.3c00013)
Supplement: Supplementary file 1 — pg3c00013_si_001.pdf [file pg3c00013_si_001.pdf]

Supporting Information

Temperature-Dependent Intensity Modulated Two-Photon Excited Fluorescence Microscopy for High Resolution Mapping of Charge Carrier Dynamics

Qi Shi†, Pushpendra Kumar‡, and Tõnu Pullerits†*

† The Division of Chemical Physics and NanoLund, Lund University, Box 124, 22100 Lund, Sweden

‡ Department of Physics, Kirori Mal College, University of Delhi, Delhi-110007, India

AUTHOR INFORMATION

Corresponding Authors

*Tonu.Pullerits@chemphys.lu.se

S1. Optical setup

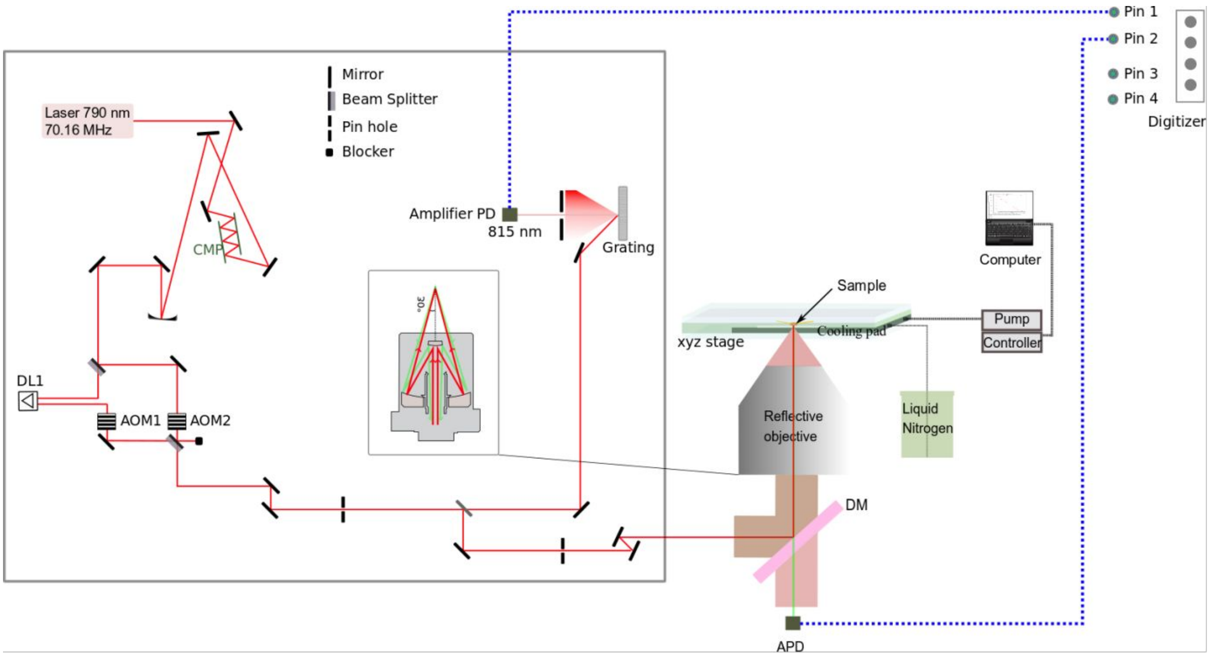

Figure S1, the schematic of setup of phase modulation photoluminescence microscope, with OS (Oscillator), CMP (Chirp mirror pair), DM (Dichroic mirror), BS (Beam splitter), AOM (Acoustic-optic modulator), M (Microscope), APD (Avalanche photodiode), and (b) the inverted microscope (Nikon Ti-S), with RO (Reflective objective). The original frequency of oscillator is 379 THz. The operating frequencies of two AOM are 55 MHz and 54.95 MHz, respectively.

Figure S1 illustrates the optical setup used in this work. A mode locked oscillator (OS) of Ti-Sa laser is used as the light source. Broad band laser pulses were with the spectrum width from 700 nm to 900 nm and the pulse duration of about 10 fs. Due to the group velocity dispersion induced by the optical

elements in the whole setup, a pair of chirp mirror pairs (CMP) is used to compensate the group velocity. A time dependent phase change was added in each arm of a Mach-Zehnder interferometer by two acousto-optic modulators (AOM) to modulate the average intensity of laser beam. The driven frequencies of radio wave in the AOM are 55 MHz and 54.95 MHz respectively with the difference of 50 KHz. After the third beam splitter, one part is used as the reference which can reflect the fluctuations in the laser intensity. Another part is sent to an inverted microscope (M). A dichroic mirror is equipped in the microscope to reflects light longer than 650 nm and transmits the shorter wavelengths. A reflective objective (RO) with a numerical aperture of 0.65 is used to tightly focus the laser beam onto the sample. The intensity of two-photon induced photoluminescence (PL) is detected by an avalanche photodiode (APD). The bandwidth of APD is about 5 MHz. A temperature-controlled stage (Linkam Scientific Instruments, LTS420E-P) was used to vary the temperatures of sample at 298, 273, 253, 233, 213, 193, 173, 153, 133 and 113 K to explore the phase transition of sample. The temperature values are rounded in this work. A scanner (MAD CITY LABS INC, MCLS02957) is used to scan the sample film in 2 μm scale. Since the two-photon absorption depends on the square of the intensity, the excitation is smaller than the focal spot of the laser beam. The two-photon excited photocurrent map with a μm spatially resolution is established based on the setup in our previous work[1].

SEM image

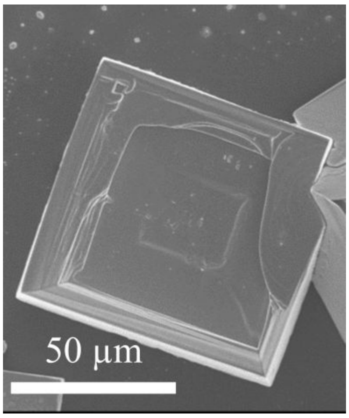

Figure S2 The SEM image of the $MAPbBr_3$ perovskite crystals.

S2. Excitation fluence calculation

The focus diameter d in the two-photon microscopy with the relective objective NA=0.5 is:

$$d = 1.22\frac{\lambda}{NA} = 1.22 * \frac{790\ nm}{0.5} = 1.927\ (\mu m)$$

The excitation fluence I is:

$$I = \frac{10\ mW}{\pi r^2} = \frac{10\ mW}{\pi r^2} = \frac{10\ mW}{2.9166 * 10^{-8} cm^2} = 3.4286 * 10^5 \frac{W}{cm^2}$$

The transition of photons per cm^3 per pulse is:

$$1W = 1\frac{J}{s} = \frac{1}{2.5177 * 10^{-19}}\frac{\mathrm{pho}ton}{s} = 3.972 * 10^{18}\frac{\mathrm{pho}ton}{s} = 5.66 * 10^{10}\frac{\mathrm{pho}ton}{pulse}$$

$$R = \beta I^2 = 10\,\frac{\mathrm{cm}}{GW} * \left(3.4286 * 10^5 \frac{W}{cm^2}\right)^2 = 1175.5\,\frac{W}{cm^3} = 6.655 * 10^{13} \frac{excitons}{cm^3 * pulse}$$

R is the amount of excitation by one pulse. And the two photon absorption coefficient β is cited from our previous work[2].

The excitation density calculated here $6.655 * 10^{13} \frac{photon}{cm^3 * pulse}$ indicated the predominant first-order recombination process in $MAPbBr_3$ film compared with the reported results[3,4]. The averaged distance between two photons is about 114.5 nm, and this is close to the reported diffusion lengths 100 $\pm$ 50 nm of solution processed $MAPbBr_3$ film[5].

S3 Accumulation effect in the first order recombination process[6]

The effect of the accumulation effect can be described as following:

$\mathrm{P}(\sigma)$ describes the population of systems in excited state. And the time evolution of the excited state population is given by the kinetic equation which consists of the relaxation and excitation transition with light intensity.

$$\frac{d(\mathrm{P}(\sigma))}{d\sigma} = -\Gamma \mathrm{P}(\sigma) + R[1 - \mathrm{P}(\sigma)] \sum_n a_n \delta(\sigma - \mathrm{n}), \mathrm{P}(0) = 0 \tag{S1}$$

where $\sigma \equiv \frac{t}{t_0}$ counts the exciting laser pulses and t_0 is the interval between the consecutive pulses. The first term at the right-hand side of (S1) describes the population decay bringing the system back to the ground state. $\Gamma = \frac{t_0}{\tau}$ is the amount of decay during the pulse interval and τ is the population depletion time which is equal to the population depletion time. The second term describes the probability of two-photon excitation by the intensity modulated pulses with $a_n = (1 + \cos(\Omega t))^2$, n refers to a pulse in the laser pulse train, and the delta function warrants that $t = n * t_0$. R is the amount of excitation per pulse ($6.655 * 10^{13} \frac{\mathrm{photon}}{cm^3 * pulse}$). And $[1 - \mathrm{P}(\sigma)]$ accounts for the depletion of the ground state due to the accumulation.

The Fourier transform of the solution of kinetic equation (9) can be performed as:

$$\mathcal{F}[\mathrm{P}(\sigma)](\omega) = \frac{1}{2\pi} \int_{-\infty}^{\infty} \mathrm{P}(\sigma) e^{-i\omega\sigma} d\sigma \tag{S2}$$

In the steady state approximation, the Taylor expansion is performed.

$$\mathcal{F}[\mathrm{P}(\sigma)](\omega) = \frac{R_0 (1 - \gamma e^{-i\omega})}{2\pi} \frac{\Gamma - i\omega}{\Gamma^2 + \omega^2} \sum_{-\infty}^{\infty} \left(a_n - \beta {a_n}^2 + \beta^2 {a_n}^3 + \mathrm{O}[a_n]^4\right) e^{-i\omega n} \tag{S3}$$

$$\mathcal{F}[\mathrm{P}(\sigma)](\omega) = R_0\left(1-\gamma e^{-i\tilde{\Omega}}\right)\frac{\Gamma - i\tilde{\Omega}}{\Gamma^2+\tilde{\Omega}^2}\left(1-\frac{7}{2}\beta+\frac{99}{8}\beta^2\right)\delta(\tilde{\Omega}-\omega) + \frac{R_0}{4}\left(1-\gamma e^{-i2\tilde{\Omega}}\right)\frac{\Gamma - i2\tilde{\Omega}}{\Gamma^2+4\tilde{\Omega}^2}\left(1 - 7\beta + \frac{495}{16}\beta^2\right)\delta(2\tilde{\Omega}-\omega) - \frac{\beta R_0}{2}\left(1-\gamma e^{-i3\tilde{\Omega}}\right)\frac{\Gamma - i3\tilde{\Omega}}{\Gamma^2+9\tilde{\Omega}^2}\left(1-\frac{55}{8}\beta\right)\delta(3\tilde{\Omega}-\omega) - \frac{\beta R_0}{16}\left(1-\gamma e^{-i4\tilde{\Omega}}\right)\frac{\Gamma - i4\tilde{\Omega}}{\Gamma^2+16\tilde{\Omega}^2}\left(1-\frac{33}{2}\beta\right)\delta(4\tilde{\Omega}-\omega) + O(\beta^3) + \ldots$$

(S4)

where $\tilde{\Omega} = \Omega t_0$ and $\beta = \frac{\gamma R_0}{1-\gamma}$.

The modulus of the Fourier transformed complex numbers show modulation amplitudes at first, second, third and fourth harmonic frequencies generated by the first-order recombination with charge accumulation effect are denoted $AE1H$, $AE2H$, $AE3H$, and $AE4H$, respectively (**Equation S5-S8**).

$$AE1H = R_0\sqrt{\left(1+\gamma^2-2\gamma\cos(\tilde{\Omega})\right)\frac{1}{\Gamma^2+\tilde{\Omega}^2}}\left(1-\frac{7}{2}\beta+\frac{99}{8}\beta^2\right)$$

$$AE2H = \frac{R_0}{4}\sqrt{\left(1+\gamma^2-2\gamma\cos(2\tilde{\Omega})\right)\frac{1}{\Gamma^2+4\tilde{\Omega}^2}}\left(1-7\beta+\frac{495}{16}\beta^2\right)$$

$$AE3H = \frac{\beta R_0}{2}\sqrt{\left(1+\gamma^2-2\gamma\cos(3\tilde{\Omega})\right)\frac{1}{\Gamma^2+9\tilde{\Omega}^2}}\left(1-\frac{55}{8}\beta\right)$$

$$AE4H = \frac{\beta R_0}{16}\sqrt{\left(1+\gamma^2-2\gamma\cos(4\tilde{\Omega})\right)\frac{1}{\Gamma^2+16\tilde{\Omega}^2}}\left(1-\frac{33}{2}\beta\right)$$

(S5-S8)

The phase of the Fourier transformed complex numbers show phase at first, second, third and fourth harmonic frequencies generated by the first-order recombination with charge accumulation effect are denoted ϕ_{A1H}, ϕ_{A2H}, $\phi_{A3H}$and ϕ_{A4H}, respectively (**Equation S9-S12**).

$$\phi_{A1H} = 2\arctan\left(\frac{-\tilde{\Omega}+\gamma\tilde{\Omega}\cos(\tilde{\Omega})+\gamma\Gamma sin(\tilde{\Omega})}{\Gamma-\gamma\Gamma cos(\tilde{\Omega})+\gamma\tilde{\Omega}\sin(\tilde{\Omega})+\sqrt{(\Gamma^2+\tilde{\Omega}^2)(1+\gamma^2-2\gamma cos(\tilde{\Omega}))}}\right)$$

$$\phi_{A2H} = 2\arctan\left(\frac{-2\tilde{\Omega}+2\gamma\tilde{\Omega}\cos(2\tilde{\Omega})+\gamma\Gamma sin(2\tilde{\Omega})}{\Gamma-\gamma\Gamma cos(2\tilde{\Omega})+2\gamma\tilde{\Omega}\sin(2\tilde{\Omega})+\sqrt{(\Gamma^2+4\tilde{\Omega}^2)(1+\gamma^2-2\gamma cos(2\tilde{\Omega}))}}\right)$$

$$\phi_{A3H} = 2\arctan\left(\frac{-3\tilde{\Omega}+3\gamma\tilde{\Omega}\cos(3\tilde{\Omega})+\gamma\Gamma sin(3\tilde{\Omega})}{\Gamma-\gamma\Gamma cos(3\tilde{\Omega})+3\gamma\tilde{\Omega}\sin(3\tilde{\Omega})-\sqrt{(\Gamma^2+9\tilde{\Omega}^2)(1+\gamma^2-2\gamma cos(3\tilde{\Omega}))}}\right)$$

$$\phi_{A4H} = 2\arctan\left(\frac{-4\tilde{\Omega}+4\gamma\tilde{\Omega}\cos(4\tilde{\Omega})+\gamma\Gamma sin(4\tilde{\Omega})}{\Gamma-\gamma\Gamma cos(4\tilde{\Omega})+4\gamma\tilde{\Omega}\sin(4\tilde{\Omega})-\sqrt{(\Gamma^2+16\tilde{\Omega}^2)(1+\gamma^2-2\gamma cos(4\tilde{\Omega}))}}\right)$$

(S9-S12)

And the amplitude ratios as:

$$R_{21} = R_{\frac{AE2H}{AE1H}} = \frac{1}{4}\sqrt{\frac{\Gamma^2 + 4\tilde{\Omega}^2(1+\gamma^2 - 2\gamma\cos(2\tilde{\Omega}))}{\Gamma^2 + \tilde{\Omega}^2(1+\gamma^2 - 2\gamma\cos(\tilde{\Omega}))}}\left(1 - \frac{7}{2}\beta + \frac{101}{16}\beta^2\right)$$

$$R_{31} = R_{\frac{AE3H}{AE1H}} = \frac{\beta}{2}\sqrt{\frac{\Gamma^2 + 9\tilde{\Omega}^2 1 - 2\gamma\cos(3\tilde{\Omega}) + \gamma^2}{\Gamma^2 + \tilde{\Omega}^2 \quad 1 - 2\gamma cos\tilde{\Omega} + \gamma^2}}\left(1 - \frac{27}{8}\beta\right)$$

$$R_{41} = R_{\frac{AE4H}{AE1H}} = \frac{\beta}{16}\sqrt{\frac{\Gamma^2 + 16\tilde{\Omega}^2 1 - 2\gamma\cos(4\tilde{\Omega}) + \gamma^2}{\Gamma^2 + \tilde{\Omega}^2 \quad 1 - 2\gamma cos\tilde{\Omega} + \gamma^2}}(1 - 13\beta)$$

(S13-15)

The simulation of the four amplitudes ($AE1H$, $AE2H$, $AE3H$, and $AE4H$), four phases (ϕ_{A1H}, ϕ_{A2H}, $\phi_{A3H}$and ϕ_{A4H}) and the three amplitude ratios (R_{21}, R_{31} and R_{41}) with different ground state recovery time (10 ns to 200 ns) are plotted in **Figure S2**.

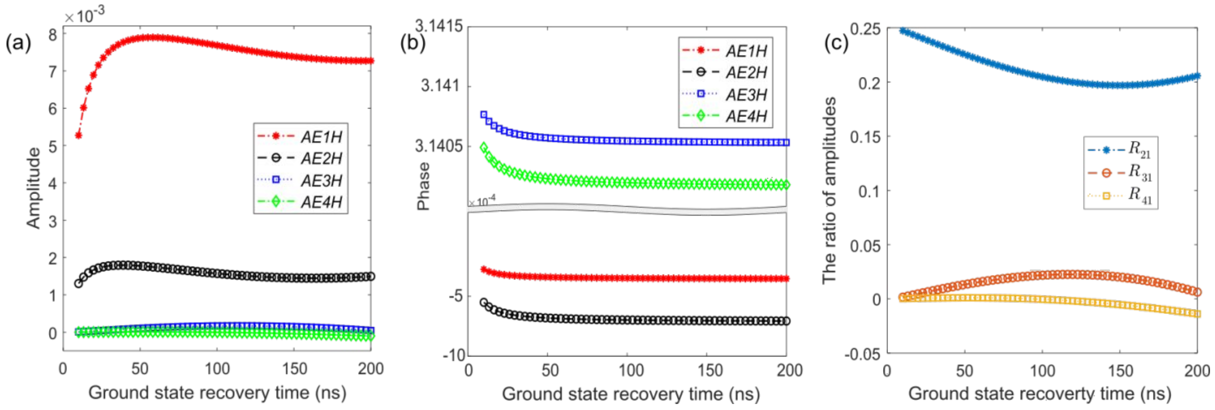

Figure S3 (a) Simulated results for the four harmonic amplitudes ($AE1H$, $AE2H$, $AE3H$, and $AE4H$) from the first-order recombination with charge accumulation effect with different population depletion time (10 ns to 200 ns). (b) Simulated results for the four harmonic phases (ϕ_{A1H}, ϕ_{A2H}, $\phi_{A3H}$and ϕ_{A4H}) from the first-order recombination with charge accumulation effect with different population depletion time (10 ns to 200 ns). (c) Simulated results for the three amplitude ratios (R_{21}, R_{31} and R_{41}) between with different population depletion time (10 ns to 200 ns). R_0 is set as 0.01.

From the figure, the four amplitudes $AE1H$, $AE2H$, $AE3H$, and $AE4H$ are increasing while the four phases ϕ_{A1H}, ϕ_{A2H}, $\phi_{A3H}$and ϕ_{A4H} are decreasing with increased population depletion time. Besides, *AE3H* and *AE4H* appear with the π phase shift (opposite sign) relative to *AE1H* and *AE2H* when the ground state recovery time is from 14.25 ns to 80 ns. Consequently, they will partially offset the *F3H&S3H* and *F4H&S4H*. While only *AE3H* appears with the π phase shift (opposite sign) relative to *AE1H, AE2H* and *AE4H* when the ground state recovery time is larger than 80 ns, meaning that *AE3H* will partially offset the *F3H&S3H.*

S4. Intensity modulation spectroscopy model simulation

The modulus of the complex number after Fourier transforms of the time domain data show modulation amplitudes at the first, second, third, and fourth harmonic frequencies denoted as **A1H**, **A2H**, **A3H**, and **A4H** at 50, 100, 150, and 200 kHz, respectively. They can be expressed as the sum of the first-order recombination (*F1H, F2H*), accumulation effect (*AE1H, AE2H, AE3H, AE4H*) and the second order recombination (*S1H, S2H, S3H, S4H*) at four harmonic frequencies, respectively.

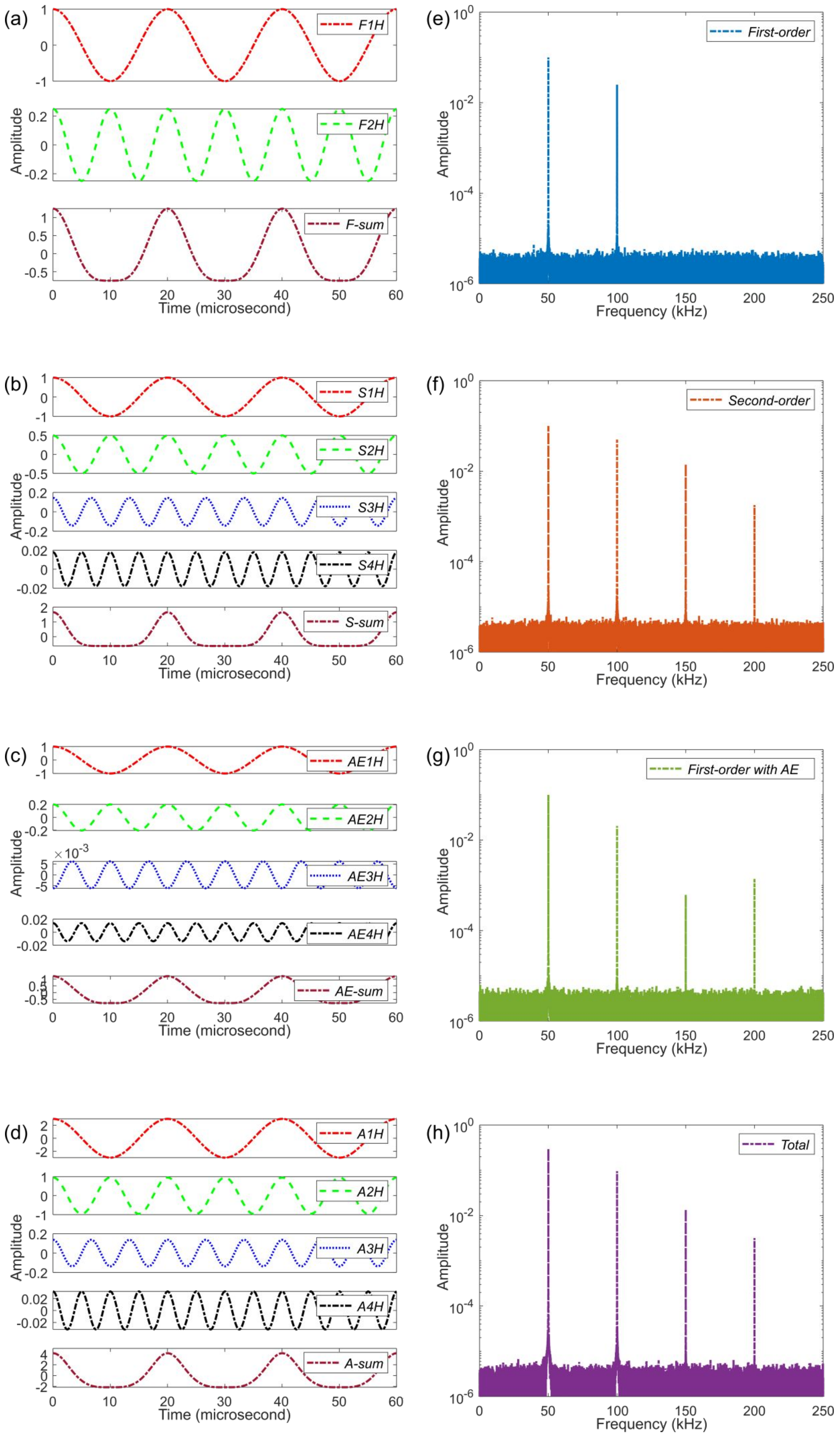
(a)
F1H
F2H
F-sum
Amplitude
Time (microsecond)
(b)
S1H
S2H
S3H
S4H
S-sum
Amplitude
Time (microsecond)
(c)
AE1H
AE2H
AE3H
AE4H
AE-sum
Amplitude
Time (microsecond)
(d)
A1H
A2H
A3H
A4H
A-sum
Amplitude
Time (microsecond)
(e)
First-order
Amplitude
Frequency (kHz)
(f)
Second-order
Amplitude
Frequency (kHz)
(g)
First-order with AE
Amplitude
Frequency (kHz)
(h)
Total
Amplitude
Frequency (kHz)

Figure S4 Simulated time-domain signals of corresponding harmonic signals and total sum signals from the first-order recombination process (a), second order recombination process (b), first order recombination process with charge accumulation effect (c), and the sum of the three processes (d). Simulated frequency-domain signals after the FFT of corresponding time-domain signals (0.1 s for 2 MSa/s sampling rate) from first-order recombination process (e), second order recombination process (f), first order recombination process with charge accumulation effect (g), and the sum of the three processes (h). The r_1, r_2 and r_{1A} are set to 1, the ratio $F1H{:}F2H$ is set as $1{:}\frac{1}{4}$, $S1H{:}S2H{:}S3H{:}S4H$is set as $1{:}\frac{1}{2}{:}\frac{1}{7}{:}\frac{1}{56}$, $AE1H{:}AE2H{:}AE3H{:}AE4H$ is set as $1{:}R_{21}{:}R_{31}{:}R_{41}$. And the amplitudes are added 1 to avoid the negative values. The transition rate R_0 is set to 0.01 and the population depletion time is set as 200 ns. A small noise was added before operating FFT to avoid the interference.

S5. The relative errors of A1H, A2H, A3H and A4H for four-character regions performance at different initial given time scales and temperatures.

Figure S4 shows the relationships between the initial ground state recovery time (the starting value for the iterative Trust-Region fitting procedure) and the results (Fitted time scale (ns)) at 300 K for the four characteristic regions. It turns out that in all four regions the convergence of τ is very robust and independent on the initial guess. Therefore 14.25 ns was used as the starting value of τ fitting in all pixels.

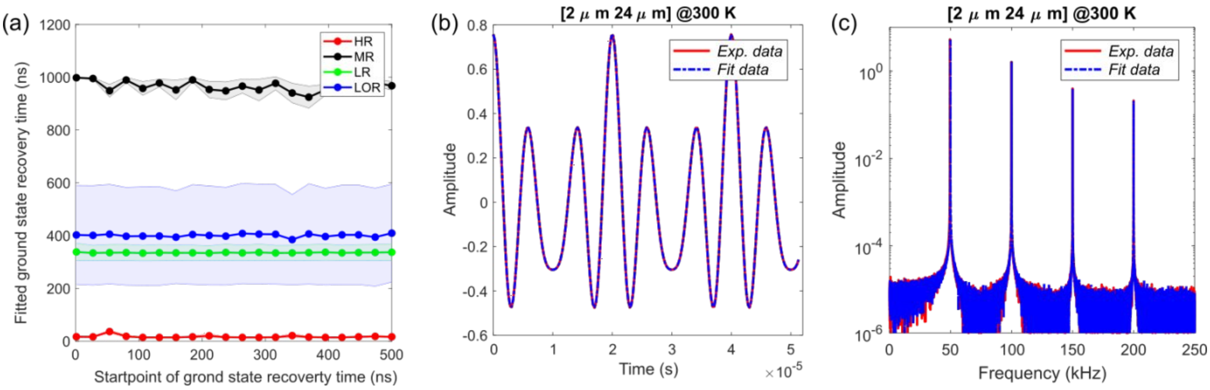

Figure S5 (a) The relationships between the Startpoint of ground state recovery time used in the fitting and the resulting time at 300 K taken separately for four characteristic regions. The shaded regions represent the 0.5*SD (standard deviation). **(b)** Time-domain signals of experimental data (position [2 µm, 24 µm] at 300 K) with red line vs the fitted results with blue dotted line. **(c)** Frequency-domain signals after the FFT of corresponding time-domain signals (0.1 s for 2 MSa/s sampling rate) of experimental data with red line vs the fitted results with blue dotted line. A small noise was added before operating FFT to avoid the interference effect.

A thorough analyses of the relative errors of the fitting parameters in the four regions is provided in the supplementary material. The relative errors for **A1H**, **A2H** and **A3H** are small, while the larger relative error of **A4H** at low temperature probably originates from the fluctuation of the cooling panel in the temperature control system. Since A4H is weak compared to other harmonics, the error for this amplitude does not significantly influence the following analyses.

The relative error between the experimental amplitudes **A1H**, **A2H**, **A3H** and **A4H** and the corresponding model amplitudes can be expressed as,

$$R_{err-1H} = \frac{A1H_{fit} - \mathbf{A1H}}{\mathbf{A1H}},$$

$$R_{err-2H} = \frac{A2H_{fit} - \mathbf{A2H}}{\mathbf{A2H}},$$

$$R_{err-3H} = \frac{A3H_{fit} - \mathbf{A3H}}{\mathbf{A3H}},$$

$$R_{err-4H} = \frac{A4H_{fit} - \mathbf{A4H}}{\mathbf{A4H}}.$$

(S16-S19)

These relative errors for four regions at different given initial time scale are presented in **Figure S5**. And these relative errors for four regions at different temperatures are presented in **Figure S6**.

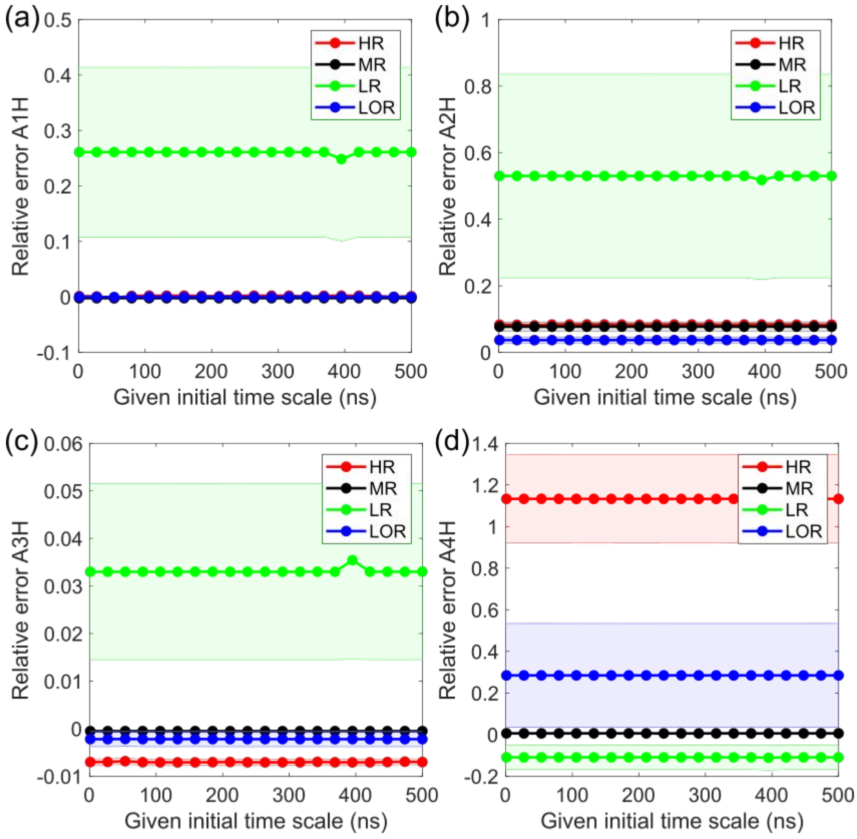

Figure S6 (a-d) the relative errors of high, medium, low, and localized spot regions (HR, MR, LR, and LOR) at different given initial time scales. The enclosed pink, purple, and light blue rectangles represent the relative errors of A1H (R_{err-1H}), A2H (R_{err-2H}), A3H (R_{err-3H}), and A4H (R_{err-4H}), respectively.

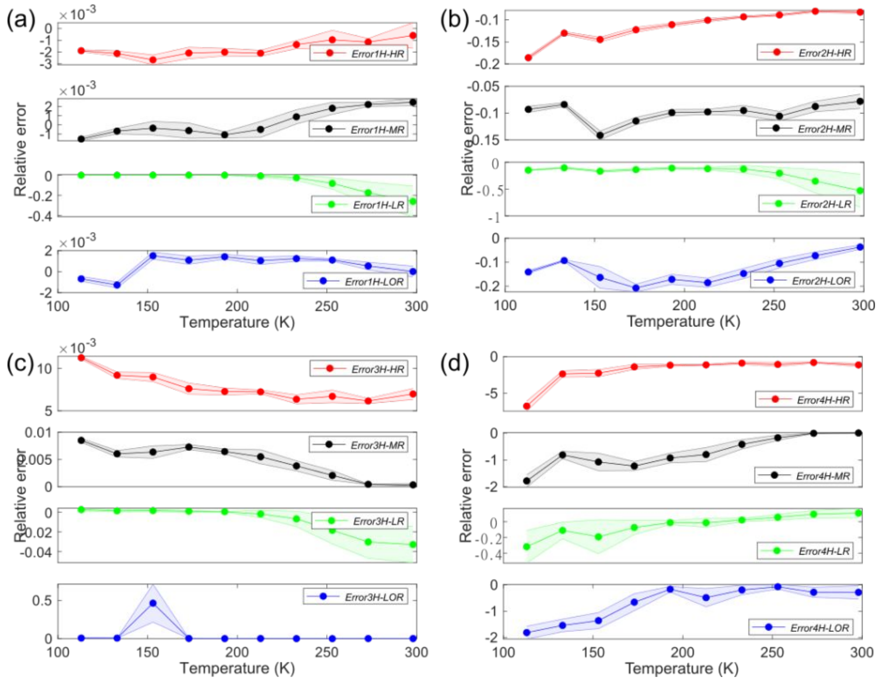

Figure S7 (a-d) the relative errors of high, medium, low, and localized spot regions (HR, MR, LR, and LOR) at different temperatures. The enclosed pink, purple, and light blue rectangles represent the relative errors of A1H (R_{err-1H}), A2H (R_{err-2H}), A3H (R_{err-3H}), and A4H (R_{err-4H}), respectively.

S6. Temperature dependence of r_1, r_2, r_{1A}, and τ in the localized spot region

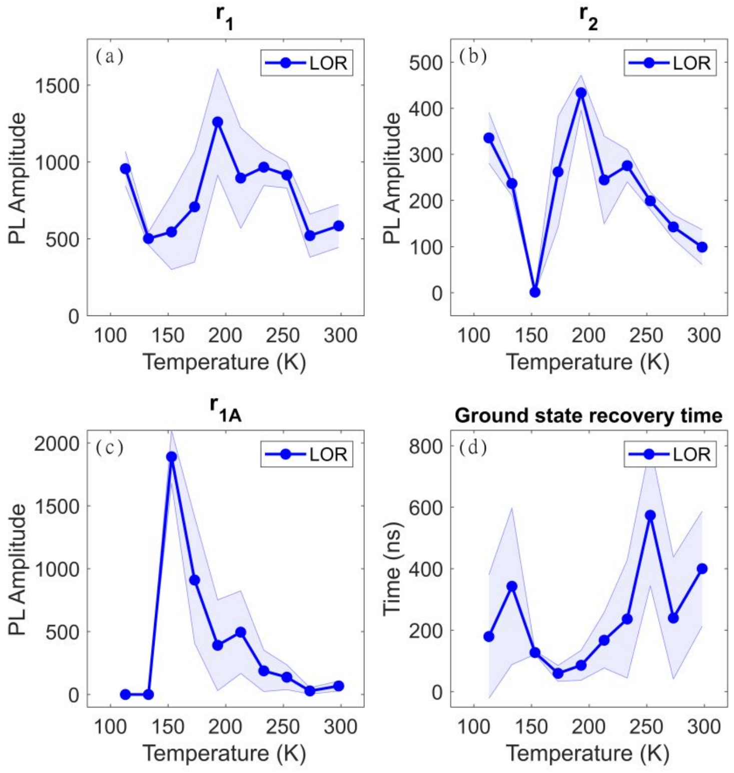

Figure S8 Temperature dependence of (a) first-order PL ($\boldsymbol{r_1}$), (b) second-order PL ($\boldsymbol{r_2}$), (c) accumulation effect PL ($\boldsymbol{r_{1A}}$), and (d) the averaged ground state recovery time τ of accumulation effect in the localized spot region. The shaded regions represent the 0.5*SD. The SD are calculated based on the 9, 16, 22, and 6 pixels in localized spot regions (LOR).

For the emission behaviors in localized spot region, charge carriers recombine more complicate. It is observed the first-order and second-order PL emission (r_1 & r_2) (Figure 5 a-b) for the localized spot region increase from 300 K to 190 K (stage 1), then decrease from 190 K to 150 K (stage 2), and last increase from 150 K to 110 K (stage 3), which are closely related to the morphology shapes in three stages (Figure S8). The increase of r_1 & r_2 in stage 1&3 could cause by the similar reasons that PL increase in these two relatively stable morphology geometries. While the decrease of r_1 & $r_2$in stage 2 indicate the morphology change hinder the first-order and second-order PL emission (r_1 & r_2) a lot.

The decreased ground state recovery time and the increased r_{1A} amplitude (Figure 5 c-d) in the tetragonal phase with decreased temperature may be due to the increased small spot effect. During the phase transition from tetragonal to orthorhombic at around 150 K[7–11], the accumulation effect PL in the localized spot regions significantly decreases, indicating that the small spot effect may disappear and alternatively additional defects may be induced due to the possible morphology change.

S7. Temperature dependence of τ in the HR, MR, and LR

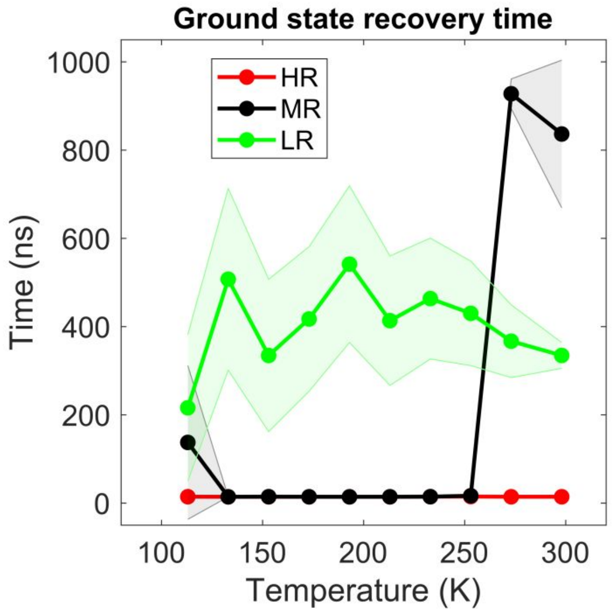

Figure S9 Temperature dependence of the averaged ground state recovery time τ of accumulation effect in the four intensity regions. The shaded area represents half of the standard deviation (SD). The SDs are calculated based on the 9, 16, and 22 pixels in the HR, MR, and LR, respectively.

S8. Summarized results of the r_1, r_2, r_{1A}, and τ for four-character regions at different temperatures

Table S1 the averaged ground state recovery time r_1, r_2, r_{1A}, and τ of the four characteristic regions at different temperatures are shown here.

		300 K	270 K	250 K	230 K	210 K	190 K	170 K	150 K	130 K	110 K
τ (ns)	HR	14	14	14	14	14	14	14	14	14	14
	MR	840	930	17	14	14	14	14	14	14	140
	LR	330	370	430	460	410	540	420	330	510	220
	LOR	400	240	570	240	170	90	60	130	340	180
r_{1A}	HR	0	0	0	0	0	0	0	0	0	0
	MR	2	3	0	0	0	0	0	0	0	0
	LR	9	9	9	9	9	7	7	7	6	10
	LOR	70	30	140	190	500	190	910	1890	0	0
r_1	HR	93	110	200	240	310	460	630	990	1410	4260
	MR	23	23	36	52	67	85	140	210	250	530
	LR	2	3	6	10	18	26	46	66	150	322
	LOR	580	520	915	966	900	1260	700	545	500	960
r_2	HR	140	170	270	320	420	600	750	1000	930	1770
	MR	20	17	37	52	62	74	105	140	150	280
	LR	0.5	1	2	4	10	11	25	33	60	97
	LOR	100	140	200	275	245	430	260	2	240	330

Table S2 the ratios between the total excitations (r_1+r_{1A}+r_2) in medium PL region, low PL region, localized spot region and high PL region at different temperatures are shown here.

	300 K	270 K	250 K	230 K	210 K	190 K	170 K	150 K	130 K	110 K
$\frac{\boldsymbol{TotalExci_{MR}}}{\boldsymbol{TotalExci_{HR}}}$	0.2	0.15	0.15	0.19	0.18	0.15	0.18	0.18	0.17	0.13
$\frac{\boldsymbol{TotalExci_{LR}}}{\boldsymbol{TotalExci_{HR}}}$	0.05	0.05	0.04	0.04	0.05	0.04	0.06	0.05	0.09	0.07

$\frac{TotalExci_{LOR}}{TotalExci_{HR}}$	3.2	2.4	2.6	2.6	2.2	2.0	1.4	1.2	0.3	0.2

S9. Modelling of the fraction of free charges over the total excited state population.

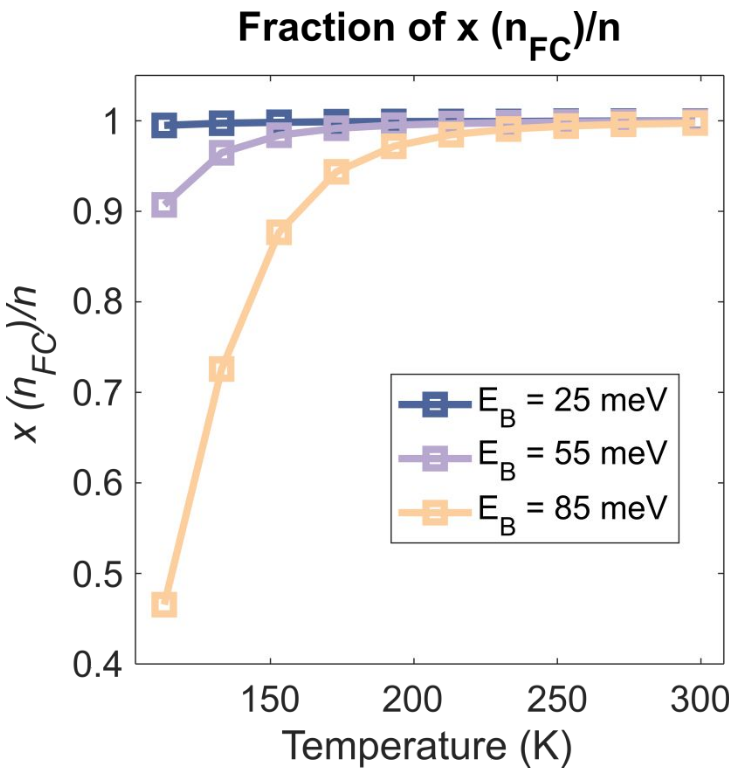

Figure S10 simulation of the ratio $x(\frac{n_{FC}}{n})$ of the free charge n_{FC} over the total excited state population n at different temperatures.

The populations of the free carriers and the excitons in a semiconductor at any given temperature is usually approximated by the Saha-Langmuir equation (1)[12–15]:

$$\frac{x^2}{1-x} = \frac{1}{n}\left(\frac{2\pi\mu k_B T}{h^2} e^{\frac{-E_B}{k_B T}}\right) \quad (20)$$

where x is the ratio between the free charge carriers over the total excitation density, n is the excitation density ($7 * 10^{13}\ cm^{-3}$), h is the Planck constant, E_b is the exciton binding energy (25, 55, and 85 meV), T is the temperature, k_B is the Boltzmann constant and μ is the reduced mass of the exciton.

S10. Abbreviations in this paper

1H First harmonic

2H Second harmonic

3H Third harmonic

4H Fourth harmonic

A1H 1H from first order recombination with and without charge accumulation effect, and second order recombination

A2H 2H from first order recombination with and without charge accumulation effect, and second order recombination

A3H 3H in from first order recombination with and without charge accumulation effect, and second order recombination

A4H 4H in from first order recombination with and without charge accumulation effect, and second order recombination

AE1H 1H from first-order recombination with charge accumulation effect

AE2H 2H from first-order recombination with charge accumulation effect

AE3H 3H from first-order recombination with charge accumulation effect

AE4H 4H from first-order recombination with charge accumulation effect

AOM Acoustic-optical modulator

APD Avalanched photodiode

DFT Discrete Fourier transform

CMP Chirped mirror pair

DFT Discrete Fourier transform

DM Dichroic mirror

F1H 1H from first-order recombination

F2H 2H from first-order recombination

F3H 3H from first-order recombination

F4H 4H from first-order recombination

FFT Fast Fourier transform

HR High PL region

LED light-emitting diodes

LOR Localized spot region

LR Low PL region

M Microscope

MR Medium PL region

MZI March-Zehnder interferometer

OR	Orthorhombic
PL	Photoluminescence
PC	Photocurrent
PV	Photovoltaic
RO	Reflective objective
r_1	First-order PL emission
r_{1_Ex}	First -order PL emission from the excitons
r_{1A}	First-order with accumulation effect PL emission
r_{1A_eh}	First-order PL emission with charge accumulation from the electron-hole pairs
r_{1_eh}	First-order PL emission from the electron-hole pairs α_{eh} ($= \alpha_C + \alpha_{free}$)
r_2	Second-order PL emission
r_{2_eh}	Second-order PL emission from electron-hole pairs
SFR	Second-to-first order ratio
S1H	1H from second-order recombination
S2H	2H from second -order recombination
S3H	3H from second -order recombination
S4H	4H from second -order recombination
SL	Saha-Langmuir
TE	Tetragonal
VB	Valence band
τ	Ground state recovery time

References

(1) Bin Yang, Junsheng Chen, Qi Shi, Zhengjun Wang, M. G.; Alexander Dobrovolsky, Ivan G. Scheblykin, Khadga Jung Karki, Keli Han, T. P. High Resolution Mapping of Two-Photon Excited Photocurrent in Perovskite Microplate Photodetector. *J. Phys. Chem. Lett.* **2018**, *9* (17), 5017−5022.

(2) Chen, J.; Žídek, K.; Chábera, P.; Liu, D.; Cheng, P.; Nuuttila, L.; Al-Marri, M. J.; Lehtivuori, H.; Messing, M. E.; Han, K.; Zheng, K.; Pullerits, T. Size-And Wavelength-Dependent Two-Photon Absorption Cross-Section of CsPbBr3 Perovskite Quantum Dots. *J. Phys. Chem. Lett.* **2017**, *8* (10), 2316–2321. https://doi.org/10.1021/acs.jpclett.7b00613.

(3) Sum, T. C.; Mathews, N.; Xing, G.; Lim, S. S.; Chong, W. K.; Giovanni, D.; Dewi, H. A. Spectral Features and Charge Dynamics of Lead Halide Perovskites: Origins and Interpretations. *Accounts of Chemical Research* **2016**, *49* (2), 294–302. https://doi.org/10.1021/acs.accounts.5b00433.

(4) Zheng, K.; Zhu, Q.; Abdellah, M.; Messing, M. E.; Zhang, W.; Generalov, A.; Niu, Y.; Ribaud, L.; Canton, S. E.; Pullerits, T. Exciton Binding Energy and the Nature of Emissive States in

Organometal Halide Perovskites. *J. Phys. Chem. Lett.* **2015**, *6* (15), 2969–2975. https://doi.org/10.1021/acs.jpclett.5b01252.

(5) Kedem, N.; Brenner, T. M.; Kulbak, M.; Schaefer, N.; Levcenko, S.; Levine, I.; Abou-Ras, D.; Hodes, G.; Cahen, D. Light-Induced Increase of Electron Diffusion Length in a p-n Junction Type CH3NH3PbBr3 Perovskite Solar Cell. *Journal of Physical Chemistry Letters* **2015**, *6* (13), 2469–2476. https://doi.org/10.1021/acs.jpclett.5b00889.

(6) Osipov, V. Al; Shang, X.; Hansen, T.; Pullerits, T.; Karki, K. J. Nature of Relaxation Processes Revealed by the Action Signals of Intensity-Modulated Light Fields. *Phys. Rev. A* **2016**, *94* (5), 053845. https://doi.org/10.1103/PhysRevA.94.053845.

(7) Wang, K. H.; Li, L. C.; Shellaiah, M.; Sun, K. W. Structural and Photophysical Properties of Methylammonium Lead Tribromide (MAPbBr3) Single Crystals. *Sci Rep* **2017**, *7* (1), 1–14. https://doi.org/10.1038/s41598-017-13571-1.

(8) Chen, Q.; De Marco, N.; Yang, Y.; Song, T. Bin; Chen, C. C.; Zhao, H.; Hong, Z.; Zhou, H.; Yang, Y. Under the Spotlight: The Organic-Inorganic Hybrid Halide Perovskite for Optoelectronic Applications. *Nano Today* **2015**, *10* (3), 355–396. https://doi.org/10.1016/j.nantod.2015.04.009.

(9) Stoumpos, C. C.; Malliakas, C. D.; Kanatzidis, M. G. Semiconducting Tin and Lead Iodide Perovskites with Organic Cations: Phase Transitions, High Mobilities, and near-Infrared Photoluminescent Properties. *Inorg Chem* **2013**, *52* (15), 9019–9038. https://doi.org/10.1021/ic401215x.

(10) Guo, D.; Bartesaghi, D.; Wei, H.; Hutter, E. M.; Huang, J.; Savenije, T. J. Photoluminescence from Radiative Surface States and Excitons in Methylammonium Lead Bromide Perovskites. *J. Phys. Chem. Lett.* **2017**, *8* (17), 4258–4263. https://doi.org/10.1021/acs.jpclett.7b01642.

(11) Shi, Q.; Ghosh, S.; Kumar, P.; Folkers, L. C.; Pal, S. K.; Pullerits, T.; Karki, K. J. Variations in the Composition of the Phases Lead to the Differences in the Optoelectronic Properties of MAPbBr3 Thin Films and Crystals. *Journal of Physical Chemistry C* **2018**, *122* (38), 21817–21823. https://doi.org/10.1021/acs.jpcc.8b06937.

(12) Cingolani, R.; Calcagnile, L.; Colí, G.; Rinaldi, R.; Lomoscolo, M.; DiDio, M.; Franciosi, A.; Vanzetti, L.; LaRocca, G. C.; Campi, D. Radiative Recombination Processes in Wide-Band-Gap II–VI Quantum Wells: The Interplay between Excitons and Free Carriers. *Journal of the Optical Society of America B* **1996**, *13* (6), 1268. https://doi.org/10.1364/JOSAB.13.001268.

(13) Saha, M. N. On a Physical Theory of Stellar Spectra. *Proceedings of the Royal Society A: Mathematical, Physical and Engineering Sciences* **1921**, *99* (697), 135–153. https://doi.org/10.1098/rspa.1921.0029.

(14) D'Innocenzo, V.; Grancini, G.; Alcocer, M. J. P.; Kandada, A. R. S.; Stranks, S. D.; Lee, M. M.; Lanzani, G.; Snaith, H. J.; Petrozza, A. Excitons versus Free Charges in Organo-Lead Tri-Halide Perovskites. *Nat Commun* **2014**, *5* (1), 1–6. https://doi.org/10.1038/ncomms4586.

(15) Ghosh, S.; Shi, Q.; Pradhan, B.; Kumar, P.; Wang, Z.; Acharya, S.; Pal, S. K.; Pullerits, T.; Karki, K. J. Phonon Coupling with Excitons and Free Carriers in Formamidinium Lead Bromide Perovskite Nanocrystals. *J. Phys. Chem. Lett.* **2018**, *9* (15), 4245–4250. https://doi.org/10.1021/acs.jpclett.8b01729.
